# Large-scale analogue quantum simulation using atom dot arrays

**DOI:** 10.1038/s41586-025-10053-7

**Published:** 2026-02-04

**Authors:** M. B. Donnelly, Y. Chung, R. Garreis, S. Plugge, D. Pye, M. Kiczynski, J. Támara-Isaza, M. M. Munia, S. Sutherland, B. Voisin, L. Kranz, Y. L. Hsueh, A. M. Saffat-Ee Huq, C. R. Myers, R. Rahman, J. G. Keizer, S. K. Gorman, M. Y. Simmons

**Affiliations:** 1 Silicon Quantum Computing Pty. Ltd., UNSW Sydney, Sydney, New South Wales Australia; 2 Centre of Excellence for Quantum Computation and Communications Technology, School of Physics, UNSW Sydney, Sydney, New South Wales Australia; 3 School of Physics, UNSW Sydney, Sydney, New South Wales Australia

**Keywords:** Quantum simulation, Quantum information

## Abstract

In pursuit of a practical quantum advantage^1^, analogue quantum systems provide an invaluable way to simulate the physics of quantum materials^2–4^, quantum systems out of equilibrium^5,6^ or interaction-induced localization^7^. Notable recent progress to realize such systems has been achieved in ultracold atoms^8–12^, superconducting circuits^13–15^ and twisted van der Waals materials^16–19^. However, so far, these platforms have struggled to simulate large-scale strongly interacting fermionic systems at low temperatures, at which electronic correlations dominate materials properties and numerical simulations remain restricted in accuracy and scope^20,21^. Here we demonstrate the realization of a new platform consisting of large-scale 2D arrays of sub-nanometre precision-engineered atom-based quantum dots (15,000 sites) to simulate strongly interacting, low-temperature physics. By observing a metal–insulator (MI) transition on a 2D square lattice of atom-based quantum dots, we demonstrate independent and precise control of the on-site interaction *U* and tunnelling *t*. Magneto-transport measurements further indicate the formation of an insulating state driven by Mott–Hubbard/Anderson physics and promising signatures of correlated electron physics. These precision-engineered analogue quantum simulators provide a unique platform to simulate quantum materials on arbitrary 2D lattices and to explore many unanswered questions in the formation of quantum magnetism, interacting topological quantum matter and unconventional superconductivity.

## Abstract (web summary)

A new platform comprising large-scale 2D arrays of quantum dots patterned with sub-nanometre precision, with each quantum dot defined by tens of phosphorus atoms doped into silicon, allows for analogue simulation of quantum materials on arbitrary lattices.

## Main

Strongly correlated quantum states, ubiquitous in quantum systems with complex band topology and strong interactions, are one of the most active areas of research in quantum materials and chemistry^3,20–23^. Analogue quantum simulations are a useful tool for investigating these systems, particularly in regimes in which the applicability of numerical techniques is limited. For different simulator platforms^1,8–19^, figures of merit include the electron bandwidth and interaction strength, temperature and the number of simulated lattice sites. Their use is further underscored by the ability to realize distinct lattice geometries, on-site degrees of freedom and by the physical observables that are accessible to experimental measurement.

Recent works on topological states in 1D chains^24^ and Fermi–Hubbard physics in small 2D arrays^25^ have shown that atom-based quantum dots, precision-manufactured using scanning tunnelling microscope (STM) lithography^26,27^ (Supplementary Information Section S1A), have many unique qualities advantageous to analogue quantum simulation. The strong Coulomb potential of the donor nuclei naturally creates strong local and long-range interactions that play a key role in many complex phenomena in quantum materials and chemistry and the precision and flexibility of STM lithography enables the patterning of arbitrary quantum dot and lattice geometries. Furthermore, atom-based quantum dots do not require confinement electrodes as in gate-defined quantum dots, creating a simpler system that is amenable to scaling up to thousands of lattice sites. These unique qualities of atom-based quantum dot arrays make them attractive for reaching simulation regimes that are challenging to access for other platforms such as gate-defined quantum dots^28–30^, ultracold atoms^8–11^ and van der Waals materials^16–19^, in which physical limitations in temperature^12^ and lattice geometry^16^ reduce the scope of systems that can be simulated. So far, atom-based quantum dot arrays have not yet been demonstrated at a scale useful for analogue quantum simulation owing to the challenge of maintaining sub-nanometre precision control in STM fabrication over the scale of micrometres.

**Fig. 1:**
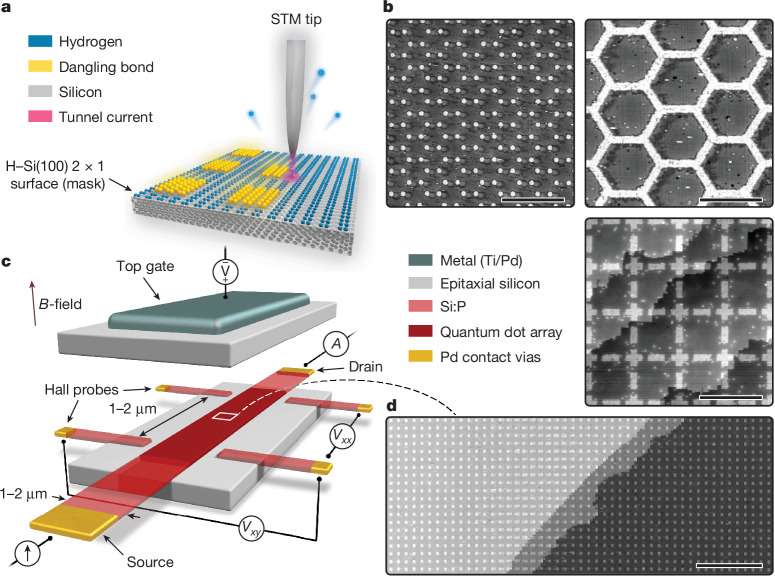
Large-scale quantum simulators using precision-engineered atom-based quantum dots in silicon. **a**, Illustration of the STM hydrogen lithography technique used to fabricate the quantum dot arrays. An atomically sharp metallic tip scans a hydrogen-terminated silicon surface and selectively removes individual hydrogen atoms to create a lithographic mask of dangling silicon bonds. Subsequent phosphine dosing is used to selectively dope the lithographic region, embedding arbitrary 2D geometries of atom-based quantum dots. **b**, Examples of STM-defined lattices: hexagonal lattice with circular quantum dots (top left); honeycomb structure (top right); Lieb lattice with rectangular and cross-shaped quantum dots (bottom right). **c**, Schematic of the quantum analogue simulator. The STM-defined array and Hall probes are encapsulated in about 80 nm of epitaxial silicon and a metallic top gate (Ti/Pd) is patterned directly over the array on the silicon surface. Charge-transport measurements are performed by applying a current or voltage through the source/drain contacts and reading out the two-point or four-point voltages at the source/drain or Hall contacts. **d**, A large-scale STM image of a square-lattice array, showing about 700 out of its 15,000 quantum dots. The terraces visible in the image have no impact on the physics of the dot array, as they are removed when the device is encapsulated in epitaxial silicon. Scale bars, 100 nm (**b** top left, **d**); 50 nm (**b** top right); 40 nm (**b** bottom right).

**Fig. 2:**
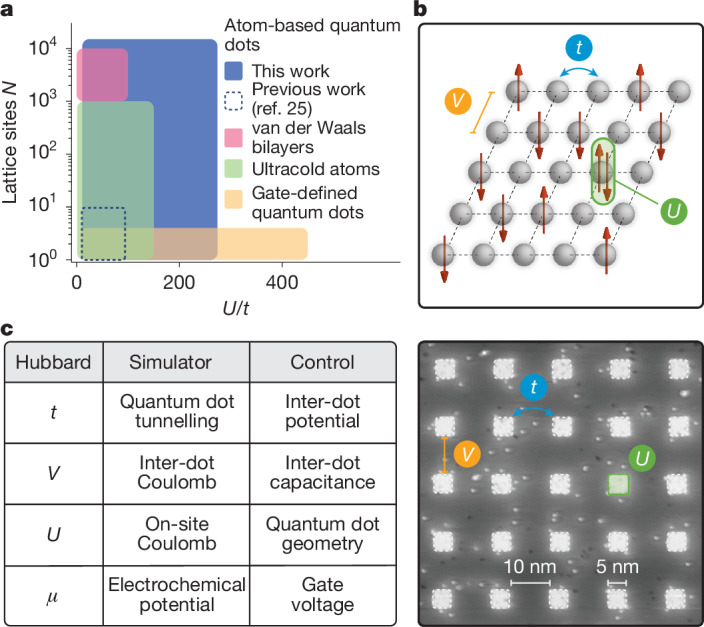
Simulation of strongly interacting physics on a 2D square-lattice quantum dot array. **a**, Approximate range of the interaction strength *U*/*t* and number of lattice sites *N* in different platforms of analogue quantum simulation. For atom-based quantum dots, we plot the range for both previous 2D simulations^25^ and this work. **b**, Top, schematic of a Hubbard model on a 2D square lattice. The sites (grey) hold up to two electrons (red arrows) with electron hopping terms *t* and on-site (inter-site) electron–electron interaction *U* (*V*). Bottom, zoomed-in image of a quantum dot array showing the equivalent Hubbard parameters realized in our quantum simulators; see also Fig. 1. **c**, The energy term in the Hubbard model, the corresponding quantity in the quantum analogue simulator and the respective method of control.

**Fig. 3:**
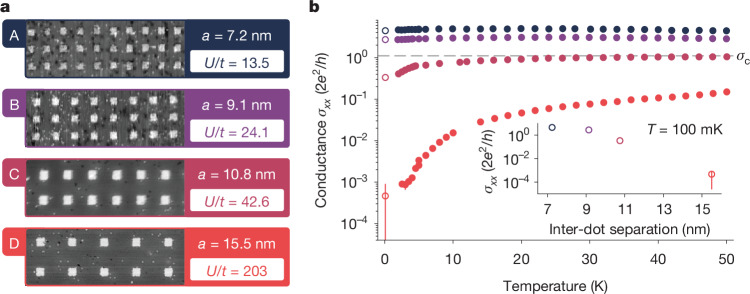
Engineering *U*/*t* across the MI transition. **a**, Scanning tunnelling micrographs that show close-up sections of the lithographic masks used to fabricate the quantum dot arrays. For arrays A–D (top to bottom), the inter-dot distance is increased, reducing the tunnel coupling. The predicted interaction strengths *U*/*t* are as indicated. **b**, Temperature dependence of the conductance *σ*_*xx*_, exhibiting metallic behaviour for arrays A and B and a weak and strong insulating behaviour in arrays C and D, respectively. Open circles indicate that the conductance at base temperature (0.1 K) decreases exponentially as the inter-dot distance *a* is increased (see inset). Error bars obtained from fits to *I*–*V* curves are smaller than the symbols for all but one data point. The horizontal dashed line indicates the critical conductance *σ*_c_ of the MI transition predicted for these four devices.

**Fig. 4:**
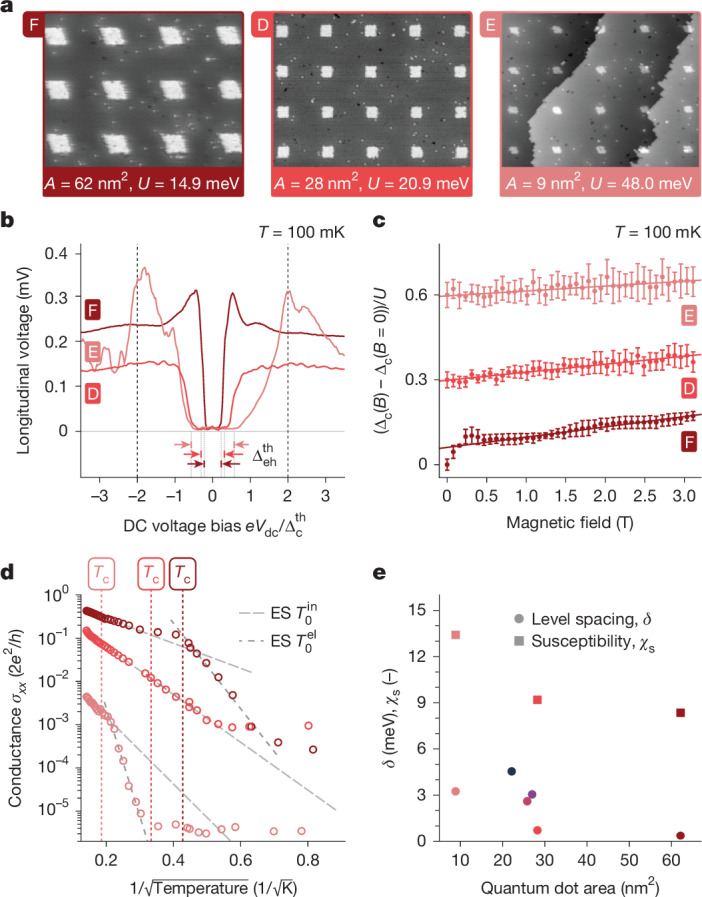
Investigating the interaction-driven nature of the insulating state. **a**, STM micrographs of arrays F, D and E (left to right), with inter-dot separation *a* ≈ 15 nm and distinct quantum dot areas *A*. The predicted interaction strength *U* is shown for each array. **b**, Bias spectroscopy for the three arrays, with bias voltage *V*_dc_ normalized by the Mott–Hubbard gap ${\Delta }_{{\rm{c}}}^{{\rm{th}}}$. A total depletion of charge transport occurs in the Coulomb gap ${\Delta }_{{\rm{eh}}}^{{\rm{th}}}$, indicated by the coloured arrows. The stronger-interacting devices D and E show a coherence peak at the Mott–Hubbard value $e{V}_{{\rm{dc}}}\approx \pm 2{\Delta }_{{\rm{c}}}^{{\rm{th}}}$, indicated by the vertical dashed lines, whereas device F exhibits one just outside the Coulomb gap. **c**, Increase of the charge gap Δ_c_ in an applied magnetic field, normalized by the interaction *U*. The linear dependence suggests electron exchange as the underlying mechanism; an effective Landé factor *g*_eff_ is extracted from a linear fit for each device. The data are offset by 0.3 for better visibility. **d**, Thermal activation of the low-bias conductance *σ*_*xx*_ in the three arrays. Transport is driven by incoherent or coherent electron co-tunnelling, with a switch at the cross-over scale *T*_c_ indicated by the vertical dashed lines. In both regimes, the conductance follows an Efros–Shklovskii (ES) law and depends exponentially on the inverse square root of temperature, but with distinct activation temperatures ${T}_{0}^{{\rm{in}},{\rm{el}}}$ (slopes of fitted dashed grey lines). **e**, The quantum dot level spacing *δ* (in meV) and the spin susceptibility *χ*_s_ (dimensionless), extracted from analysis of the data in Figs. 3b and 4c, versus the quantum dot area *A*.

In this work, we greatly expand the quantum simulation capabilities of atom-based quantum dots in terms of both precision and size (see Figs. 1 and 2a) and simulate a MI transition driven by Mott–Hubbard and Anderson physics in a series of 2D quantum dot arrays, each containing 15,000 atom-based quantum dots on a 100 × 150 square lattice. This array size eclipses the previous largest reported arrays of about ten quantum dots^24,25^ and reflects a notable step up in STM-based atomic fabrication. To achieve uniformity across such large arrays, in both the size of and the spacing between the atom-based quantum dots, we incorporated and optimized the use of an advanced STM controller (Zyvex Labs) that can correct for piezo creep and hysteresis across micron-sized areas. This method allows us to pattern arbitrary lattice geometries, as shown in Fig. 1b,d. The quantum dot array is integrated into a fully epitaxial Hall bar architecture to enable charge-transport measurements (Fig. 1c and Supplementary Information Section S4). Here the source/drain and Hall probes are also patterned using STM lithography, yielding metallic, highly phosphorus-doped silicon leads^31^. Ohmic contacts to the leads along with a metallic (Ti/Pd) global top gate for electrostatic control are added using semiconductor processing techniques. The behaviour of the system is determined by the energy scales engineered into the lattice (see Fig. 2b,c): the on-site interaction *U*_*i*_ (energy cost to add/remove an electron on site *i*), inter-site interaction *V*_*ij*_ (cost to add an electron on site *i* as a result of one on site *j*), tunnel coupling *t*_*ij*_ (hopping energy between sites *i* and *j*) and electrochemical potential *μ*_*i*_ (single-particle energy of site *i*). For next-nearest neighbour sites, we find typical *V*_*ij*_ and *t*_*ij*_ that are 50–70% and ≱5% of their nearest neighbour values, respectively (see Supplementary Information Section S2 and ref. ^25^). Next-nearest neighbour interactions are therefore substantial, whereas next-nearest neighbour tunnelling is negligible. We simulate a MI transition by manufacturing four different quantum dot arrays (labelled A–D) with increasing inter-dot separation *a* (7.2 nm, 9.1 nm, 10.8 nm and 15.5 nm), quenching the inter-dot tunnel coupling *t* from 1.54 ± 0.28 meV to 0.10 ± 0.02 meV. The quantum dot area was kept in the range *A* = 22–28 nm^2^ corresponding to about 50 phosphorus atoms in each dot and an on-site energy *U* = 20.63 ± 0.94 meV. As a result of the increasing dot separation, the effective interaction strength *U*/*t* increases from 14 to 203. Disorder in these energies is estimated at <1 meV; see Supplementary Information Section S3D. Scanning tunnelling micrographs of sections of the lithographic masks used to define each array are shown in Fig. 3a, along with the ratio *U*/*t* calculated from a combination of continuum and atomistic modelling techniques (Supplementary Information Section S3). The collective behaviour of charge carriers in the array is first examined by measuring the longitudinal conductance *σ*_*xx*_, plotted in Fig. 3b as a function of temperature. We observe that the conductance at base temperature (approximately 100 mK) decreases exponentially with increasing inter-dot separation *a* (that is, larger *U*/*t*). Theories of transport in granular metals, in which both interactions and disorder are present^32–36^, predict a MI transition at a critical conductance *σ*_c_, determined by the interaction strength *U* and level spacing *δ* of the quantum dots, with the latter estimated independently from the high-temperature conductance in each sample (Supplementary Information Section S4 and Fig. 4e). We obtain a value of the critical conductance ${\sigma }_{{\rm{c}}}\approx 1.11\frac{2{e}^{2}}{h}$, indicated by the dashed line in Fig. 3b. Consistent with this prediction, arrays A and B are metallic, whereas arrays C and D show weakly and strongly insulating low-temperature behaviour, respectively.

To further examine the nature of the observed insulating state, we manufactured two more quantum dot arrays with inter-dot separations *a* ≃ 15.1 nm and 17.1 nm (similar to the insulating array D in Fig. 3) but changing the quantum dot area *A* to 9 nm^2^ and 62 nm^2^ (arrays E and F, respectively; Fig. 4a). Electron interactions are weaker in larger quantum dots and stronger in smaller quantum dots, allowing us to control the interaction energy scales *U* and *V* by changing the quantum dot area *A* (Supplementary Information Section S3A,B). Figure 4a shows close-up STM micrographs of the lithographic masks used to define the three insulating arrays, with the corresponding dot area *A* and interaction strength *U* indicated. For a Mott–Hubbard system in the strongly interacting limit, theory predicts a single-particle charge gap of the order ${\Delta }_{{\rm{c}}}^{{\rm{th}}}\simeq \frac{1}{2}U+4V+\cdots$ (refs. ^20,21,35^) with extra terms owing to long-range interactions. Conversely, the onset of bulk charge transport in the dot arrays is governed by the Mott–Coulomb gap for electron–hole excitations ${\Delta }_{{\rm{eh}}}^{{\rm{th}}}=U-c{g}_{{\rm{T}}}{E}_{{\rm{eh}}}$, in which *E*_eh_ = 2*U* − *V* is the bare excitation energy cost, *g*_T_ the inter-dot conductance and *c* ≈ 0.281 (refs. ^35,37^) (see Supplementary Information Section S4C). The bias spectroscopy measurements in Fig. 4b are taken at base temperature (*T* ≈ 100 mK), for which we use the longitudinal voltage measured between the Hall probes as a proxy for the presence of current-carrying states in the array. For arrays E and D, these indicate a hard insulating gap at low bias within the Coulomb gap, $e|{V}_{{\rm{dc}}}|\le {\Delta }_{{\rm{eh}}}^{{\rm{th}}}$, and a coherence peak (boost of spectral weight) with subsequent saturation of the signal as the source/drain bias overcomes the Mott–Hubbard excitation energy (at $e{V}_{{\rm{dc}}}=\pm 2{\Delta }_{{\rm{c}}}^{{\rm{th}}}$ in Fig. 4b, with *V*_dc_ = *V*_S_ − *V*_D_ the total source–drain bias). For the largest-dot array F, we find a coherence peak just above the Coulomb gap ${\Delta }_{{\rm{eh}}}^{{\rm{th}}}$ and only faint features at the Mott–Hubbard scale ${\Delta }_{{\rm{c}}}^{{\rm{th}}}$ (Supplementary Information Section S4C,D). We attribute the different behaviours of the devices to the weaker interactions, smaller dot level spacing and larger inter-dot conductance going from devices E, D to F. The bias spectroscopy data show that the strength of interactions in our devices can be tuned using the size of the quantum dots.

Next we subject the insulating devices to a perpendicular magnetic field, for which we observe a distinct interaction effect in quantum dots: the enhancement of the charge addition energy (and hence charge gap) owing to the electron exchange mechanism^38^. Here electron spin states in an applied magnetic field are split by a large Zeeman energy that can be framed in terms of an interaction-enhanced Landé *g*-factor *g*_eff_. The measurements of the field-enhanced gap for devices D, E and F are in shown in Fig. 4c, with *g*_eff_ extracted from the linear fit indicated by the solid lines (for the full field-dependent voltage-bias spectroscopy data, see Supplementary Information Section S4D). The exchange mechanism acts locally on each quantum dot and we take *g*_eff_ ≃ *g*_0_(1 + *χ*_s_) with the bare value *g*_0_ = 2. Using the data in Fig. 4c, we can extract the ‘excess’ spin susceptibility *χ*_s_ and in Fig. 4e see that *χ*_s_ ~ *U* with increasing dot size *A* and decreasing on-site interaction strength *U* (Supplementary Information Section S4D). This scaling is consistent with predictions for granular metallic systems and quantum dots in which the wavefunction is spread across the donor sites within each individual dot^38–41^.

As well as bias spectroscopy, we take measurements of the low-bias longitudinal conductance *σ*_*xx*_ in devices D, E and F, shown in Fig. 4d, and observe a distinct two-step thermal activation. According to the theory of granular metals (Supplementary Information Section S4A,B), a transition from inelastic to elastic co-tunnelling of electrons in the arrays is set by the cross-over scale ${k}_{{\rm{B}}}{T}_{{\rm{c}}}\approx 0.2\sqrt{\delta U}$ (refs. ^35,37,42^). Both regimes lead to a conductance described by the Efros–Shklovskii law ${\sigma }_{{\rm{ES}}}(T)\simeq {\sigma }_{0}\exp (\,-\sqrt{{T}_{0}/T})$ (refs. ^35,37,42–44^) but with different activation temperatures ${T}_{0}^{{\rm{in}}}$ and ${T}_{0}^{{\rm{el}}}$ in the inelastic and elastic co-tunnelling regimes, respectively. Our data (Fig. 4d) show that we observe this two-step thermal activation in devices F and E and that the observed transition temperature (kink in the experimental data) closely coincides with the predicted cross-over temperature *T*_c_ (vertical dashed lines). The observed activation temperatures ${T}_{0}^{{\rm{in}},{\rm{el}}}$ extracted from fits to the data are larger in the devices with stronger interactions and broadly conform with theory expectations^35,37,42^ (see Supplementary Information Section S4B).

Within the Mott–Hubbard/Anderson picture, the findings in Figs. 3 and 4 provide evidence of a MI transition and that the atom-based quantum dots in our arrays host electron states spread across the geometric area of individual dots, with wavefunction characteristics, inter-dot tunnelling and interaction energies that match design expectations. In Fig. 4e, we plot the dot level spacing and spin susceptibility extracted for the various devices, which served as important metrics to understand the underlying physics. Going forward, the ability to precision-engineer the size, shape and lattice spacing of the dots demonstrated in Fig. 1 provides an intricate level of control, to interpolate from few donors per dot to the metallic limit or to promote the impact of different electron orbitals. Likewise, we can target future devices to fall close to or far from the MI transition, while hosting coherent electrons up to a high temperature *T*_c_.

**Fig. 5:**
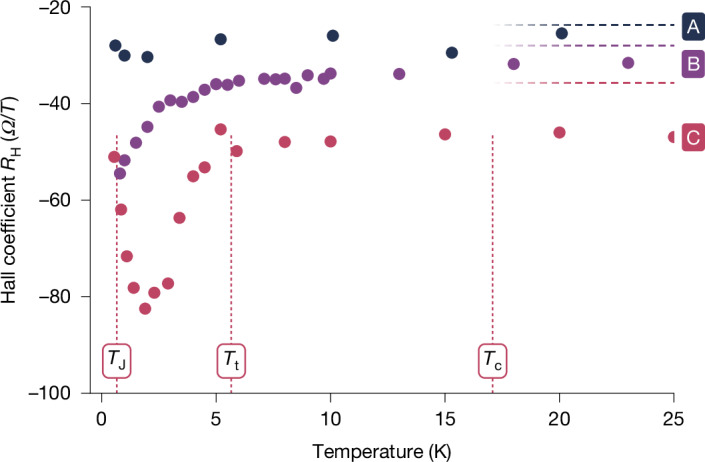
Hall coefficient data at low temperatures. The temperature-dependent Hall coefficient *R*_H_ obtained from magneto-transport measurements for the three conductive devices A–C of Fig. 3. Horizontal dashed lines denote the value expected if each donor contributes one free electron, ignoring dot lattice and interaction effects. Vertical lines indicate temperatures *T*_c,t,J_ for device C at which the physics of the electrons are predicted to undergo notable changes.

Finally, we present measurements of the temperature-dependent Hall coefficient *R*_H_, which can be used to investigate subtle changes in the nature of charge transport in interacting electron systems^16–21,33,34,45,46^. Hall coefficient data for the conductive arrays A–C of Fig. 3 are plotted in Fig. 5, with arrays D, E and F being too resistive to reliably perform Hall measurements. At temperatures >20 K, the Hall coefficient saturates at a value that, for array A, matches a non-interacting picture with carrier density *n*_H_ = 1/*eR*_H_ close to the number of dopants in the device. The dashed lines in Fig. 5 indicate the expected non-interacting Hall coefficients assuming a doping density of 2 nm^−2^ within the quantum dots. For arrays B and C, the saturated Hall coefficient reflects a lower carrier density than expected from the number of dopants, with a stronger discrepancy the larger the inter-dot spacing *a*. This is probably because of the weaker tunnelling *t*, for which some electron states in the quantum dots split off and stop contributing to transport. In the context of analogue quantum simulation, we may equate this to a trend towards band insulation or electron localization in the artificial atom lattice.

More pronounced effects are observed in the coherent tunnelling regime in the weakly insulating array C, at temperatures below *T*_c_ ≈ 17 K. We find a sharp increase in the magnitude of the Hall coefficient at temperature *T* < 6 K and a distinct turnaround at a lower *T* < 2 K. Similar trends are predicted to arise owing to Fermi-surface reconstruction for correlated electrons^33,34,45–50^ or the formation of mini-bands in moiré superlattices^16–19^. The temperature scales *T*_t_ ≈ 5.7 K and *T*_J_ ≈ 0.7 K indicated in Fig. 5 are related to the inter-dot hopping *t* and exchange coupling *J*_exc_, respectively (Supplementary Information Section S4E). Here we refrain from drawing too close a comparison with either such systems. The devices studied by us exhibit several electron states per dot, chemical potential disorder, inter-site interactions and other characteristics that may affect the Hall coefficient in unknown ways. Hence it is difficult to conclusively link the data in Fig. 5 to a Fermi-surface reconstruction^51,52^. Note that we also observe differences between forward and backward magnetic field sweeps that hint at magnetic hysteresis in devices B and C (raw data and analysis in Supplementary Information Section S2B). Yet the behaviour of the Hall coefficient below the coherence temperature *T*_c_ in device C (Fig. 5, pink) clearly differs from what is expected for a charge carrier freeze-out owing to non-correlated effects such as chemical potential disorder, for which we would find a gradual logarithmic increase of the Hall coefficient^32–37,53^. This indeed is what seems to happen in device B and has been observed in many granular metals or disordered doped semiconductors, including in continuously doped silicon phosphorus delta layers^54–56^.

In summary, we have presented a new class of analogue quantum simulators using precision-engineered atom-based quantum dots in silicon for simulating strongly interacting electron systems. Using the nanometre accuracy of STM hydrogen lithography, we pattern arrays containing 15,000 quantum dots with varying inter-dot separations (7.2 nm to 17.1 nm) and quantum dot areas (9 nm^2^ to 62 nm^2^) to simulate a Mott–Anderson MI transition. Embedding the quantum dot arrays in a Hall bar device geometry allows us to characterize their physics through a comprehensive suite of magneto-transport measurements. The observations reported here include a MI transition, the dot size dependence and exchange-interaction enhancement of the charge-transport gap, a temperature-driven cross-over from incoherent to coherent electron co-tunnelling in the insulating dot arrays and, finally, promising signatures of a Fermi-surface reconstruction witnessed by the temperature-dependent Hall coefficient. The presented data paints an encouraging picture for the use of atom-based quantum dots as a platform for large-scale analogue quantum simulation.

Having extensively tested and characterized our atom-based quantum dot arrays, we believe that this system has the potential to shed light on contemporary challenges in physics such as quantum spin liquids, interacting topological quantum matter and unconventional superconductivity. This is because of the unique capabilities of the physical platform, such as the ability to engineer large values of the interaction strengths *U* and *V* with a varying ratio *U*/*t*. As demonstrated in Fig. 1, we can implement arbitrary 2D lattice geometries as long as the dot edge length and inter-dot separation are larger than the silicon lattice constant ($a,\sqrt{A}\gg {a}_{0}=0.54\,{\rm{nm}}$) and engineer specific orbitals as valence states of individual quantum dots or multidot unit cells. This includes geometries that are hard to realize or altogether inaccessible in the ultracold atom and twisted moiré material platforms, such as the Lieb lattice or quasicrystals. An immediate next step in our experiments is the systematic exploration of electron correlation and Fermi-surface reconstruction effects hinted at by the Hall coefficient data in Fig. 5. On tuning the electron density by means of the top gate (see Fig. 1), we should be able to observe various band-filling or correlation effects^16–19^. Further, it may be possible to simulate a correlated state such as superconductivity that has very distinct transport signatures^2,3^ or to observe various kinds of magnetic order in the arrays^57,58^. Another interesting direction is the 2D Lieb lattice, as shown in Fig. 1b, with *p*-type and *d*-type ‘atomic’ orbitals on distinct sites and adjustable level detunings in the presence of strong interactions. Such a set-up mimics the Hubbard model description of a copper–oxygen plane in the cuprates, which is believed to lead to its coveted charge-transfer insulator and high-temperature superconducting states^2,3,20,21,46^.

## Online content

Any methods, additional references, Nature Portfolio reporting summaries, source data, extended data, supplementary information, acknowledgements, peer review information; details of author contributions and competing interests; and statements of data and code availability are available at 10.1038/s41586-025-10053-7.

## Supplementary information

Supplementary Information Supplementary Information Sections S1–4, including Supplementary Figs. 1–17 and Supplementary References.

## Data Availability

The raw data and code used in this article are available at Zenodo: 10.5281/zenodo.17782840 (ref. ^59^).
