# Supplementary material for: Large-scale analogue quantum simulation using atom dot arrays
Source: Nature. 2026 Feb 4;650(8102):574–9. doi: 10.1038/s41586-025-10053-7 (PMC12916296; doi:10.1038/s41586-025-10053-7)
Supplement: Supplementary file 1 — Supplementary Information Sections S1–4, including Supplementary Figs. 1–17 and Supplementary References. [file 41586_2025_10053_MOESM1_ESM.pdf]

nature portfolio

https://doi.org/10.1038/s41586-025-10053-7

Supplementary information

Large-scale analogue quantum simulation using atom dot arrays

In the format provided by the authors and unedited

Supplementary material: Large-scale analogue quantum simulation using atom dot arrays

M.B. Donnelly,[1,2] Y. Chung,[1] R. Garreis,[1] S. Plugge,[1] D. Pye,[1,2] M. Kiczynski,[1] J. Támara-Isaza,[1] M.M. Munia,[1] S. Sutherland,[1,2] B. Voisin,[1] L. Kranz,[1,2] Y.L. Hsueh,[1] A.M. Saffat-Ee Huq,[1] C.R. Myers,[1] R. Rahman,[3] J.G. Keizer,[1,2] S.K. Gorman,[1,2,*] and M.Y. Simmons[1,2,*]

[1] *Silicon Quantum Computing Pty. Ltd., UNSW Sydney, Australia*
[2] *Centre for Quantum Computing and Communication Technology, School of Physics, UNSW Sydney, Australia*
[3] *School of Physics, UNSW Sydney, Australia*

(Dated: January 26, 2026)

CONTENTS

S1. Device fabrication & measurement setup 1
A. Quantum dot array fabrication 1
B. Cryogenic charge transport measurements 2

S2. Magneto-transport characterisation of Si:P delta-layers and quantum dot arrays in the Hall bar architecture 2
A. δ-layer Hall Bar Measurements 2
B. Quantum dot array Hall bar measurements 3

S3. Modelling of the Hubbard parameters and disorder 17
A. On-site interaction U 17
B. Inter-site interactions V, V', and V'' 17
C. Electron hopping amplitudes t and t' 17
D. Disorder effects 18

S4. Charge transport theory and data analysis 19
A. High-temperature transport in granular metals 19
B. Electron co-tunnelling and thermal activation 20
C. Electron excitations and the charge transport gap 21
D. Exchange-interaction enhancement of the charge transport gap 24
E. Low-temperature transport and Hall coefficient 24

References 26

S1. DEVICE FABRICATION & MEASUREMENT SETUP

A. Quantum dot array fabrication

Manufacturing the quantum dot arrays is performed using scanning tunnelling microscopy (STM) hydrogen resist lithography [1–4], where an STM tip is used to selectively desorb hydrogen from a hydrogen terminated 2×1 reconstructed (001) silicon surface. A combined ZyVector/Nanonis STM control system is used to correct for creep and hysteresis to achieve precision lithography of the 15,000 dot array. This process is performed in an ultrahigh vacuum (UHV) system with a base pressure of $\sim 1\times10^{-11}$ mbar. After lithography the surface is dosed with phosphine gas (PH_3) at a pressure of 2×10^{-7} mbar for 2 min (18.0 Langmuir). The phosphorus atoms incorporate into the exposed silicon using a 350°C anneal for 60 s. The device is then encapsulated with $\sim$80 nm of epitaxial silicon. Standard semiconductor processing methods are used to make Ohmic contact to the buried phosphorus doped leads using palladium contacts [5] and to deposit a metallic (Ti/Pd) global top-gate.

---

* These authors contributed equally to the project supervision

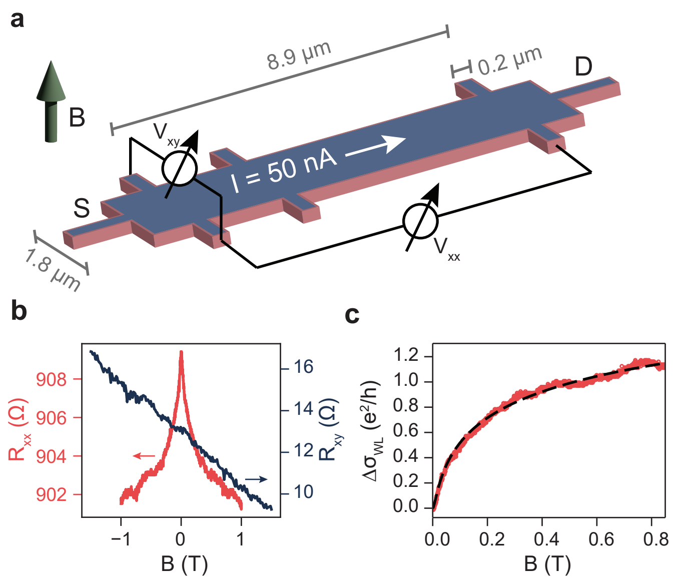

FIG. S1. **Extracting transport data and characteristics for a δ-layer Hall bar device and quantum dot arrays. a.** Schematic of the Hall bar device geometry for the δ-layer sample, cf. Fig. 1 of the main text. **b.** Longitudinal (R_{xx}) and transversal (R_{xy}) magneto-resistance calculated from the measured longitudinal (V_{xx}) and transversal (V_{xy}) voltages with a source-drain bias current $I_{\mathrm{SD}} = 50\,\mathrm{nA}$. We extract the charge carrier density ($n_{2D} = 2.510 \pm 0.011 \times 10^{14}\,\mathrm{cm}^{-2}$), the mobility ($\mu = 138.8 \pm 0.2\,\mathrm{cm}^2/\mathrm{Vs}$), and an estimate for the electron mean free path ($l = 18.00 \pm 0.03\,\mathrm{nm}$). **c.** Weak-localisation correction to the conductance extracted from b, with a Hikami fit (dashed line) for $B < 0.8\,\mathrm{T}$ used to obtain the electron phase-coherence length ($l_\phi = 226.9 \pm 1.1\,\mathrm{nm}$).

B. Cryogenic charge transport measurements

Transport measurements were performed in a dilution refrigerator with a base temperature of $\sim$100 mK. Measurements of the conductance were performed using standard low-frequency lock-in techniques with excitation frequencies of 10-20 Hz and an excitation amplitude of 0.1-0.2 mV. The current through the array was amplified by a Femto DLPCA transimpedance amplifier and subsequently measured by a lock-in amplifier. The temperature of the sample was controlled using a heater and thermometer mounted close to the sample on the dilution refrigerator coldfinger. We control the temperature using a PID loop, with the helium mixture only circulating for target temperatures between 100 mK and 20 K. The doped silicon substrate becomes conductive at a temperature of $\sim$60 K, and hence we limit the maximum temperature of the sample during experiments to 50 K ($k_B T/t \sim 10$).

S2. MAGNETO-TRANSPORT CHARACTERISATION OF SI:P DELTA-LAYERS AND QUANTUM DOT ARRAYS IN THE HALL BAR ARCHITECTURE

A. δ-layer Hall Bar Measurements

To characterise the Si:P Hall bar architecture used to measure the quantum dot arrays, we initially fabricated a device containing a nominally 0.35 ML phosphorus doped δ-layer [6] in place of the quantum dot array, see Fig. S1a. We performed magneto-transport experiments to calculate the Hall coefficient R_{H} using measurements of the Hall voltage V_{xy} (see Fig. S1b), from which the 2D charge carrier density $n_{2D} = 2.510 \pm 0.011 \times 10^{14}\,\mathrm{cm}^{-2}$, charge carrier mobility $\mu = 138.8 \pm 0.2\,\mathrm{cm}^2/\mathrm{Vs}$ and the electron mean free path $l = 18.00 \pm 0.03\,\mathrm{nm}$ were derived. We further determine the weak localisation (WL) correction to the longitudinal conductivity σ_{xx}, as shown in Fig. S1c. Fitting the Hikami formula [7] for $B < 0.8\,\mathrm{T}$ we extracted a coherence length of $l_\phi = 226.9 \pm 1.1\,\mathrm{nm}$. The characteristic values for l, l_ϕ, n_{2D}, and μ measured in the δ-doped Hall bar are in close agreement with previous published results on a much larger, hundred-micrometer scale mesa-defined Si:P δ-layer [6] (with $n_{2D} = 2.4 \pm 0.1 \times 10^{14}\,\mathrm{cm}^{-2}$, $\mu = 115 \pm 10\,\mathrm{cm}^2/\mathrm{Vs}$, $l = 23\,\mathrm{nm}$, $l_\phi = 137\,\mathrm{nm}$), demonstrating that key electrical properties can be obtained using the Hall bar architecture that is used to characterise the quantum dot arrays throughout this work.

B. Quantum dot array Hall bar measurements

The Hall coefficient data in Fig. 5, especially for the weak-insulating strongly-interacting device C, exhibits a clear non-trivial dependence of the Hall coefficient at low temperatures $T \lesssim T_c$ below the electron coherence temperature scale T_c, cf. Sec. S4 E. Curiously, we find marked differences in the fitted Hall coefficients R_H and fit offsets between backward and forward magnetic field sweeps, which is indicative of low-temperature magnetic hysteresis. For completeness, in this section we provide a thorough analysis of devices A, B, and C (device A (Fig. S2), B (Figs. S3-S9), and C (Figs. S10-S14)) related to the measured Hall coefficients noting that in the main text we focus on forward magnetic fields sweeps from one pair of Hall probes. Due to the difficulties in measuring highly resistive samples we provide a discussion on the analysis methods used to determine the data in Fig. 5 of the main text.

In the experiment, we measure the longitudinal voltage V_{xx} between two Hall-bar side contacts on the same side of the device, and the transverse voltages $V_{xy,1}$ and $V_{xy,2}$ at the two pairs of opposing Hall-bar side contacts '1' and '2', as we sweep the perpendicular magnetic field B. We also measure the source-drain current I through the Hall bar to calculate the longitudinal and transverse resistances $R_{xx} = V_{xx}/I$ and $R_{xy,1/2} = V_{xy,1/2}/I$. In our analysis of the measurement data, we account for magnetic field-antisymmetric components in the longitudinal resistance and field-symmetric components in the transverse resistances by defining the longitudinal and Hall voltages and resistances R_{Long} and R_{Hall} via

$$R_{\text{Long}}(B) = \frac{1}{2}\left[R_{xx}(B) + R_{xx}(-B)\right] + \frac{1}{2}\left[R_{xy}(B) + R_{xy}(-B)\right] \quad (1)$$

$$R_{\text{Hall}}(B) = \frac{1}{2}\left[R_{xx}(B) - R_{xx}(-B)\right] + \frac{1}{2}\left[R_{xy}(B) - R_{xy}(-B)\right] \quad (2)$$

Here R_{xy} denotes the averaged transverse resistance, that is $R_{xy} = \frac{1}{2}\left[R_{xy,1} + R_{xy,2}\right]$. The Hall coefficient R_H, plotted in Fig. 5 of the main text as a function of temperature, is taken as the gradient with magnetic field B in the low-field regime of either $R_{xy,1/2}$, R_{xy}, or R_{Hall}. In an ideal Hall bar sample all of these resistances should give equivalent results. In practice, the large resistivity and relatively small size of our samples leads to part of the source current to leak into the side contacts. Here we present the raw data and data analysis for the different devices, including for backward and forward sweeps of the applied magnetic field B. Throughout the main text we plot the forward scans for consistency across all devices. The magneto-transport measurements of device C shows strong hysteretic behaviour at low temperatures pointing to the presence of some spin magnetism effects such as ferromagnetism or non-trivial magneto-orbital effects such as the quantum anomalous Hall effect which will be the subject of further investigations. Here we discuss the raw and pre-processed magneto-transport data used to fit the Hall coefficient R_H (and other parameters) for device A, B, and C.

- **Device A - included data in main text from** $R_{xy,1}$ - Fig. S2:
  For device A, we obtained magneto-transport data for the transverse resistance $R_{xy,1}$ with one magnetic field sweep and at several temperatures $T \in [0.6, 33.0]$K. Since the device is quite conductive, it is not difficult to obtain reliable data. We do not observe drastic changes in the Hall coefficient R_H. The Hall coefficient data $R_H(T)$ from measurement in Fig. S2 (right panel) is directly reproduced in Fig. 5a of the main text.

- **Device B - included data in main text from** $R_{xy,1}$ - Figs. S3-S9:
  For device B, we obtained the full magneto-transport data for the transverse resistances $R_{xy,1/2}$ and longitudinal resistance R_{xx}, with multiple magnetic field sweeps and at several temperatures $T \in [0.5, 30.0]$K. It is our most comprehensively characterized device, partially since we observe a weak anti-localization peak in the longitudinal resistance $R_{xx}(B)$ that complicates a reliable fitting and determination of the Hall coefficient. Figs. S3 and S4 show data for a forward and backward sweep in a low-field range $B = [-0.8, 0.8]$T, with fitting and analysis in Fig. S5. Fig. S6 summarizes the fitting and analysis for these and a second set of forward and backward sweeps in the low-field range $B = [-0.8, 0.8]$T. Figs. S7 and S8 show a third set of forward and backward magnetic field sweeps up to a larger field range $B = [-3.2, 3.2]$T, and in Fig. S9 we analyze the Hall coefficients obtained from these when fitting only in the low-field window $|B| < 0.8$T (as for the first and second sets of field sweeps) versus fitting in the full available field range $|B| < 3.2$T where we find good agreement between $R_{xy,1}$ and $R_{xy,2}$.

  Between all the data sets for device B, we consistently observe a gradual down-turn in the Hall coefficient $R_H(T)$ determined from the transverse resistance $R_{xy,1}$ at low temperatures (Fig. S6 and Fig. S9, top row). In the low-field range, $R_H(T)$ determined from $R_{xy,2}$ exhibits a different behavior that is qualitatively similar across field sweeps (Fig. S6 bottom, and Fig. S9 bottom left). In Fig. 5a of the main text we show $R_H(T)$ obtained from $R_{xy,1}$ with the forward-sweep Hall coefficient shown in Fig. S6 (top left). Still we note that the non-trivial temperature dependencies and the distinct sensitivity of the transverse voltages and Hall coefficients may signal the onset of complex electron/charge transport physics in the device.

- **Device C - included data in main text from $R_{xy,2}$** - Figs. S10-S14:
  For device C, we obtained the full magneto-transport data for the transverse resistances $R_{xy,1/2}$ and longitudinal resistance R_{xx}, with one set of magnetic field sweeps and at several temperatures $T \in [0.5, 30.0]$K. Figs. S10 and S11 show data for the forward and backward sweep in the field range $B = [-0.8, 0.8]$T, respectively. Figs. S12 and S13 show the fitting and analysis of the Hall coefficient $R_H(T)$ for fits in the low-field range $|B| < 0.4$T and the full field range, respectively. We also perform an analysis of the average and difference of forward and backward sweep; this is shown in Fig. S14, and illustrates that device C exhibits some anomalous (hysteretic) contributions in the transverse Hall resistance.

  In Fig. 5a of the main text, for device C we show the forward sweep and $R_H(T)$ obtained from $R_{xy,2}$ (Fig. S12, top left). We refer to the supplementary data and mention the differences between forward and backward sweep results, and magnetic hysteresis as a possible explanation, in the main text. As for device B, the non-trivial temperature dependencies and the distinct sensitivity of the transverse voltages and Hall coefficients signals the onset of complex electron/charge transport physics at low temperatures $T < T_c \approx 17$K.

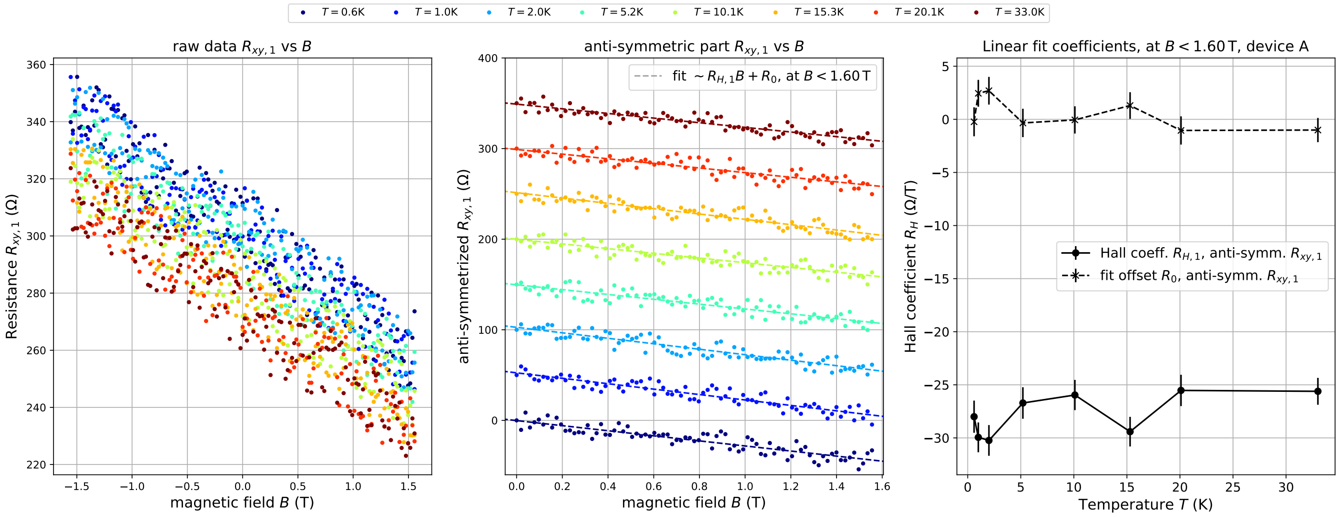

FIG. S2. **Magneto-transport measurements and Hall coefficient fits for device A.** Left panel: raw data for transverse resistance $R_{xy,1}$ measured at one pair of side-contacts of the quantum dot Hall bar device. Center panel: anti-symmetric part of $R_{xy,1}$, with $50\,\Omega$ shifts between consecutive temperatures, and a respective linear fit that determines the respective Hall coefficient $R_{H,1}$ and zero-field offset R_0. Right panel: Hall coefficient $R_{H,1}$ and zero-field offset R_0 versus temperature T, as determined from the fits in the center panel. The fitted Hall coefficient $R_H = R_{H,1}$ is shown in the main text Figure 5b.

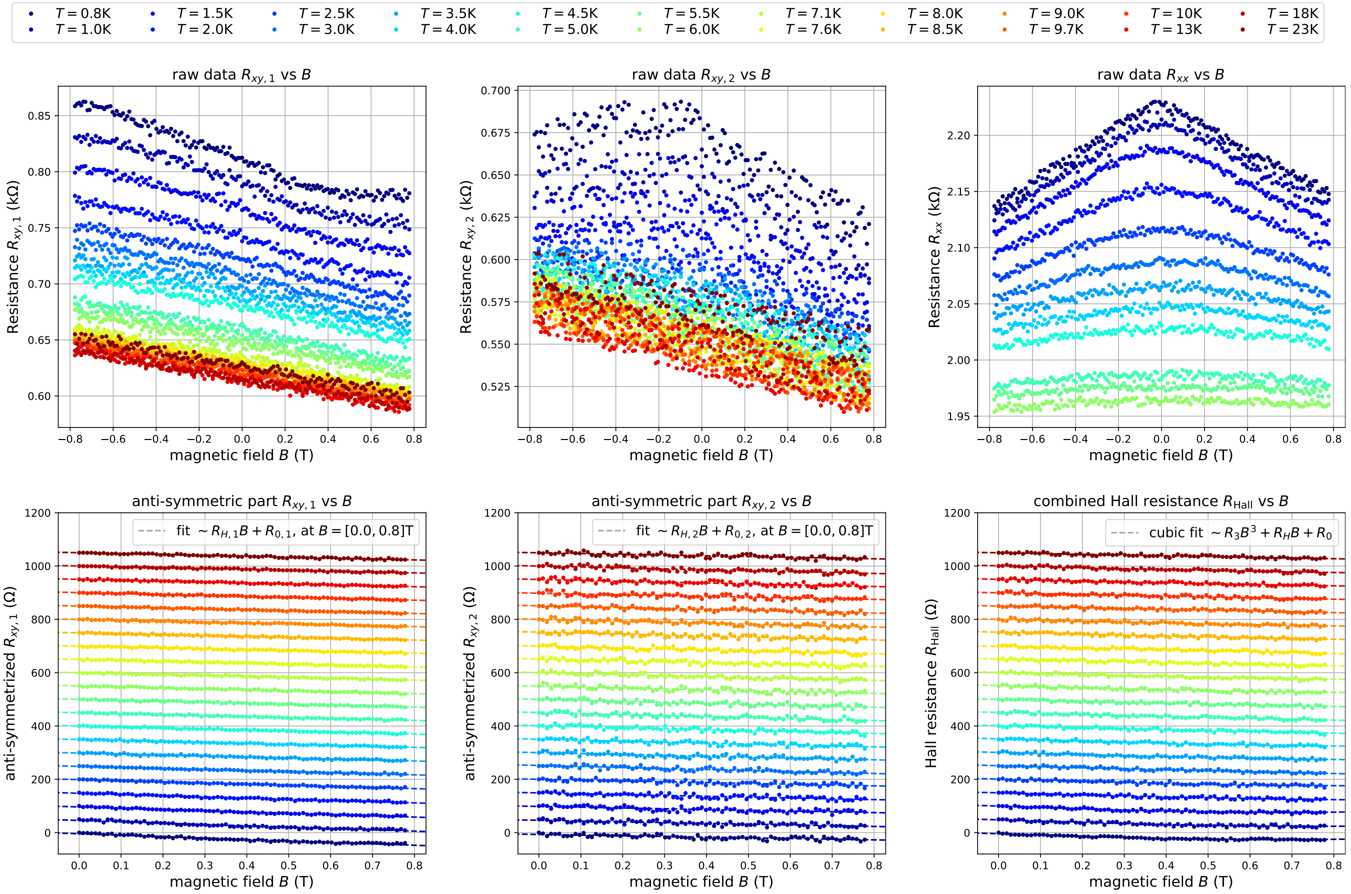

FIG. S3. **Magneto-transport measurements and Hall coefficient fits for device B [forward field sweep].** Top row: raw magneto-transport data for the transverse resistances $R_{xy,1}$ (left) and $R_{xy,2}$ (center), and the longitudinal resistance R_{xx} (right) measured during a forward sweep of the magnetic field $B = -0.8\,\mathrm{T} \to 0.8\,\mathrm{T}$ at various temperatures $T \in [0.8\,\mathrm{K}, 30.0\,\mathrm{K}]$ (see legend) for device B. Bottom row: field-antisymmetric parts of $R_{xy,1}$ (left) and $R_{xy,2}$ (center), with $50\,\Omega$ shifts between consecutive temperatures, and a linear fit (dashed lines) that determines the respective Hall coefficient $R_{H,1/2}$ and a zero-field offset $R_{0,1/2}$. The Hall resistance R_{Hall} (cf. Eq. (2); right plot, with $50\,\Omega$ shifts) is fitted by a similar linear fit or by a linear-plus-cubic polynomial in the full field range (dashed lines). Results of the fits are shown in Fig. S5.

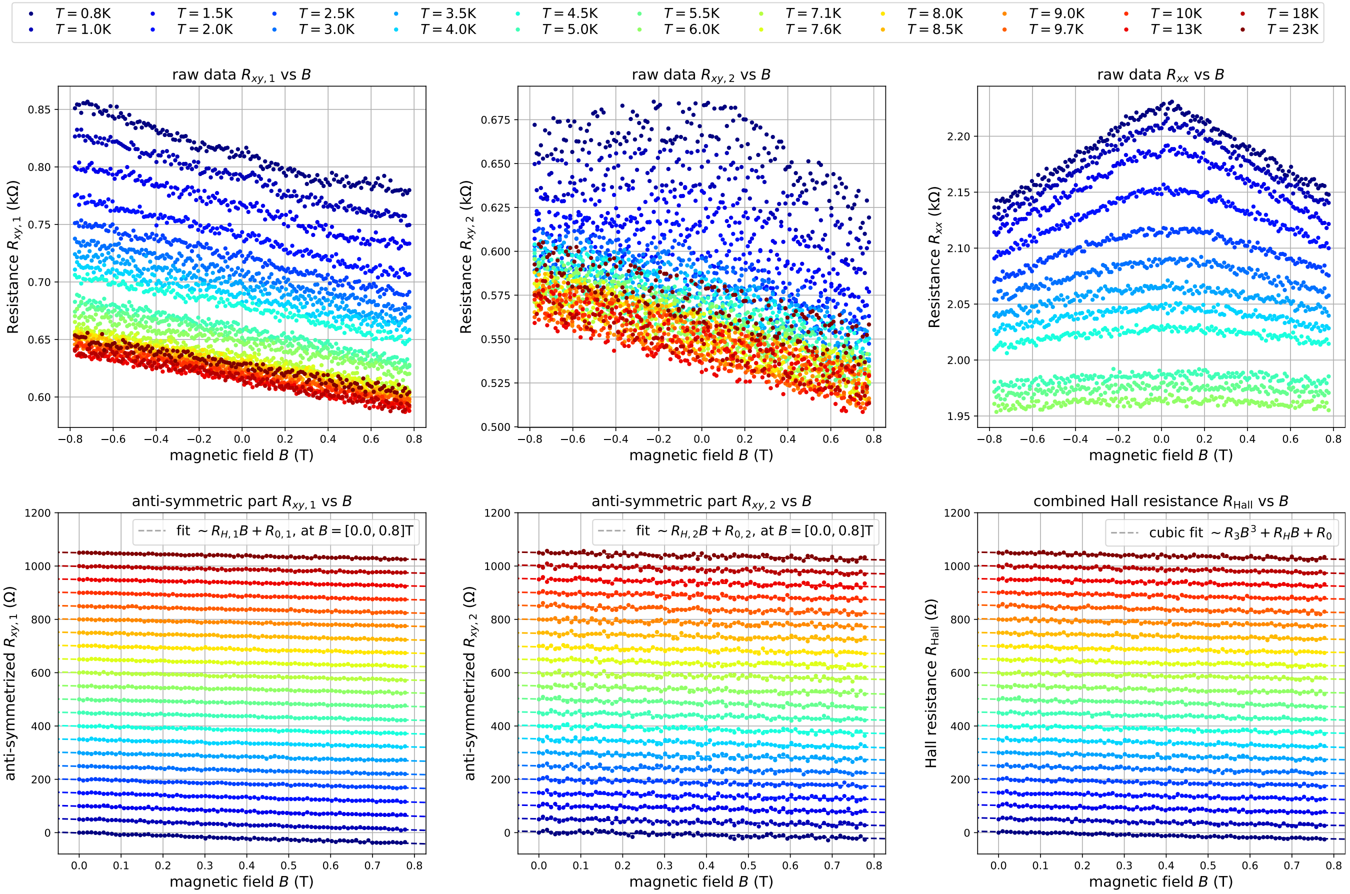

FIG. S4. **Magneto-transport measurements and Hall coefficient fits for device B [backward field sweep].** Top row: raw magneto-transport data for the transverse resistances $R_{xy,1}$ (left) and $R_{xy,2}$ (center), and the longitudinal resistance R_{xx} (right) measured during a backward sweep of the magnetic field $B = 0.8\,\mathrm{T} \to -0.8\,\mathrm{T}$ at various temperatures $T \in [0.8\,\mathrm{K}, 30.0\,\mathrm{K}]$ (see legend) for device B. Bottom row: field-antisymmetric parts of $R_{xy,1}$ (left) and $R_{xy,2}$ (center), with $50\,\Omega$ shifts between consecutive temperatures, and a linear fit (dashed lines) that determines the respective Hall coefficient $R_{H,1/2}$ and a zero-field offset $R_{0,1/2}$. The Hall resistance R_{Hall} (cf. Eq. (2); right plot, with $50\,\Omega$ shifts) is fitted by a similar linear fit or by a linear-plus-cubic polynomial in the full field range (dashed lines). Results of the fits are shown in Fig. S5.

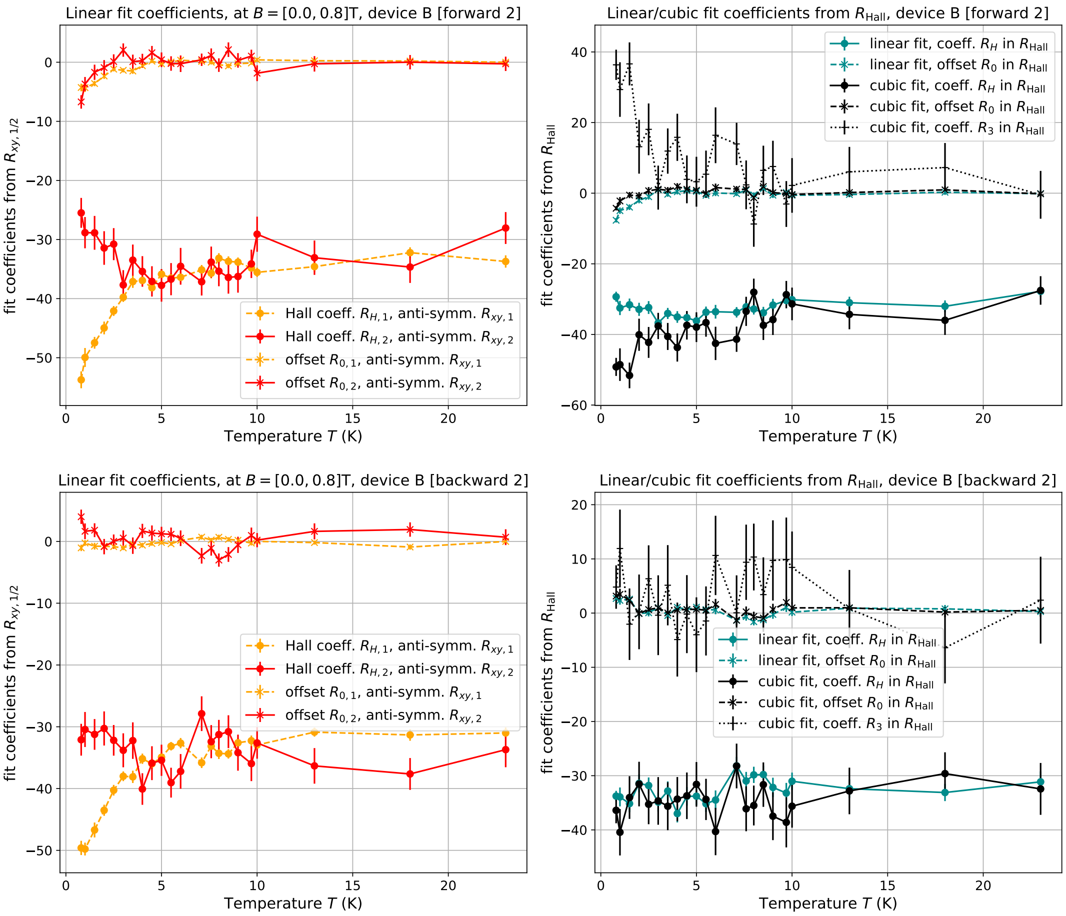

FIG. S5. **Hall coefficient and offset fit parameters for device B [forward and backward field sweeps].** Top row: Hall coefficients and offsets obtained from the forward field sweeps in Fig. S3. Fits $R_{H,1/2}$ obtained from the anti-symmetrized $R_{xy,1/2}$ (top left) show distinct behavior at low temperatures, and R_H from R_{Hall} (top right) matches the average; note that $R_{H,2}$ exhibits stronger variations/noise also visible in the raw data. The Hall coefficient $R_{H,1}$ (from $R_{xy,1}$, least noisy data) shows a flat temperature dependence above $T \approx 10\,\text{K}$, and a gradual down-turn to more negative values at $T \lesssim 8\,\text{K}$. The offsets R_0 stay close to zero. Bottom row: Hall coefficients and offsets obtained from the backward field sweeps in Fig. S4. The Hall coefficient and offset data here is broadly consistent with the forward sweep. For further detail and comparisons, see Fig. S6.

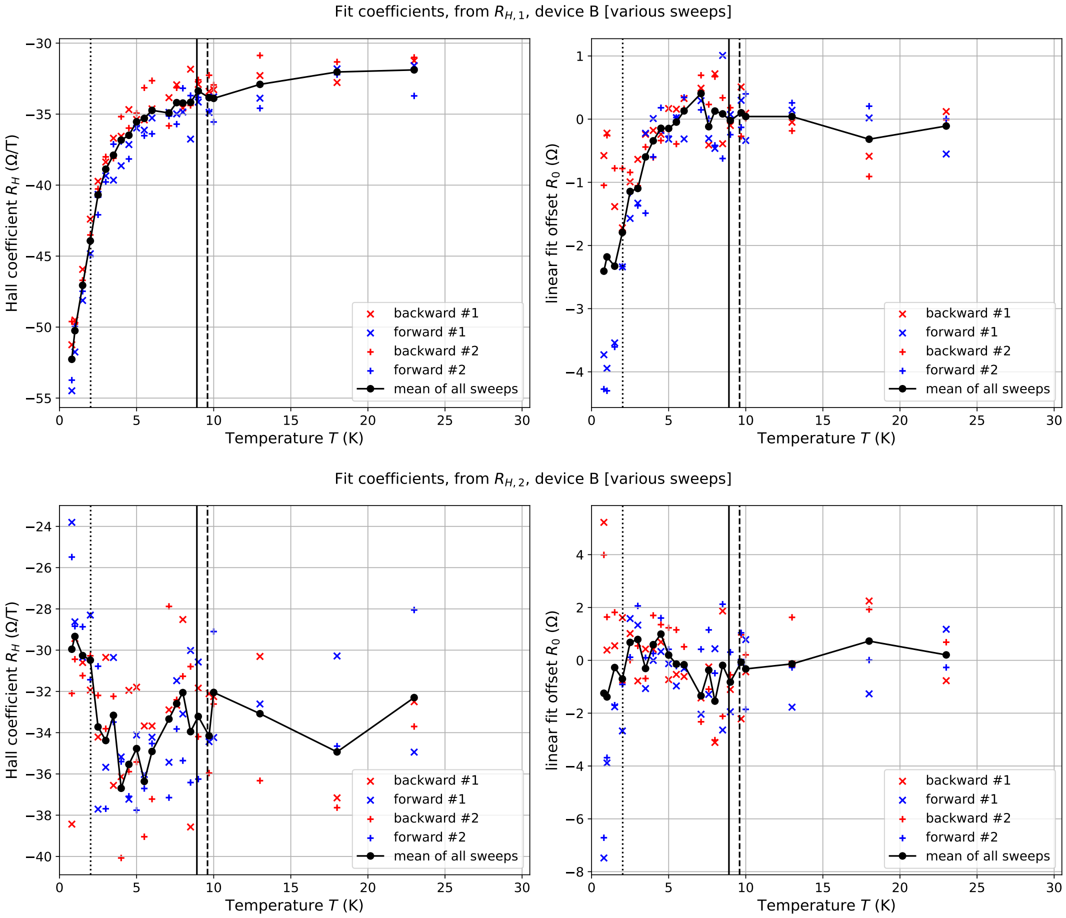

FIG. S6. **Reproducibility of the Hall coefficient R_H and offset R_0 for device B, across several field sweeps.** Top row: R_H and R_0 from fits of $R_{xy,1}$ for two sets of field sweeps as in Fig. S5. Black dots and lines indicate the average value, while red/blue symbols indicate the individual backward/forward sweep data. We find a consistent trend to a large, negative Hall coefficient at low temperatures, following roughly a logarithmic dependence as expected for a charge carrier freeze-out due to disorder effects or interactions [8–13]. The offset R_0 also grows to a finite value at low temperatures $T < T_c \approx 18\,\mathrm{K}$, with different trends dependent on sweep direction at the lowest temperatures $T \lesssim 3\,\mathrm{K}$. Bottom row: same as top row, but with coefficients from the linear fit to $R_{xy,2}$ instead. The behavior of $R_{H,2}$ is quite different from that of $R_{H,1}$ (top row), which may be attributed to the seemingly larger admixture of the longitudinal voltage/resistance $V_{xx} \sim R_{xx}$ in $R_{xy,2}$ and the more noisy raw data, as observed in Figs. S3 and S4. For the offset R_0 we nevertheless find a similar trend as in $R_{xy,1}$ (top right) for which at the lowest temperatures, the results for forward and backward sweeps diverge from each other in an apparent onset of magneto-hysteresis. The vertical dashed and dotted lines indicate the tunnelling temperature scale T_t and exchange coupling scale T_J, cf. Sec. S4 E.

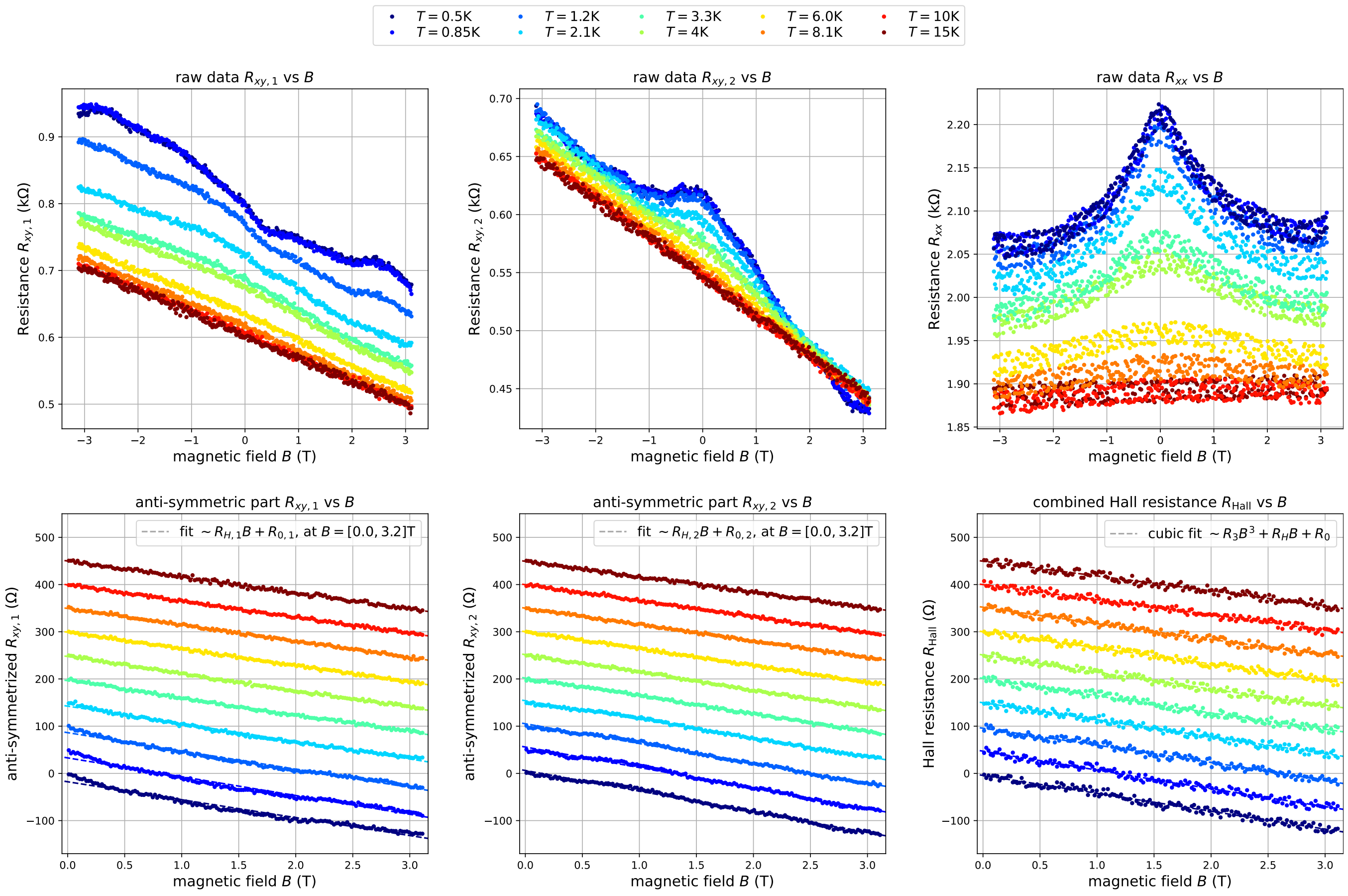

FIG. S7. **Magneto-transport measurements and Hall coefficient fits for device B [forward field sweep, large field range].** Top row: raw magneto-transport data for the transverse resistances $R_{xy,1}$ (left) and $R_{xy,2}$ (center), and the longitudinal resistance R_{xx} (right) measured during a forward sweep of the magnetic field $B = -3.2\,\mathrm{T} \to 3.2\,\mathrm{T}$ at temperatures $T \in [0.5\,\mathrm{K}, 15.0\,\mathrm{K}]$ (see legend) for device B. Bottom row: field-antisymmetric parts of $R_{xy,1}$ (left) and $R_{xy,2}$ (center), with $50\,\Omega$ shifts between consecutive temperatures, and a linear fit (dashed lines) that determines the respective Hall coefficient $R_{H,1/2}$ and a zero-field offset $R_{0,1/2}$. The Hall resistance R_{Hall} (cf. Eq. (2); right plot, with $50\,\Omega$ shifts) is fitted by a similar linear fit or by a linear-plus-cubic polynomial in the full field range (dashed lines). Results of the fits are shown in Fig. S9.

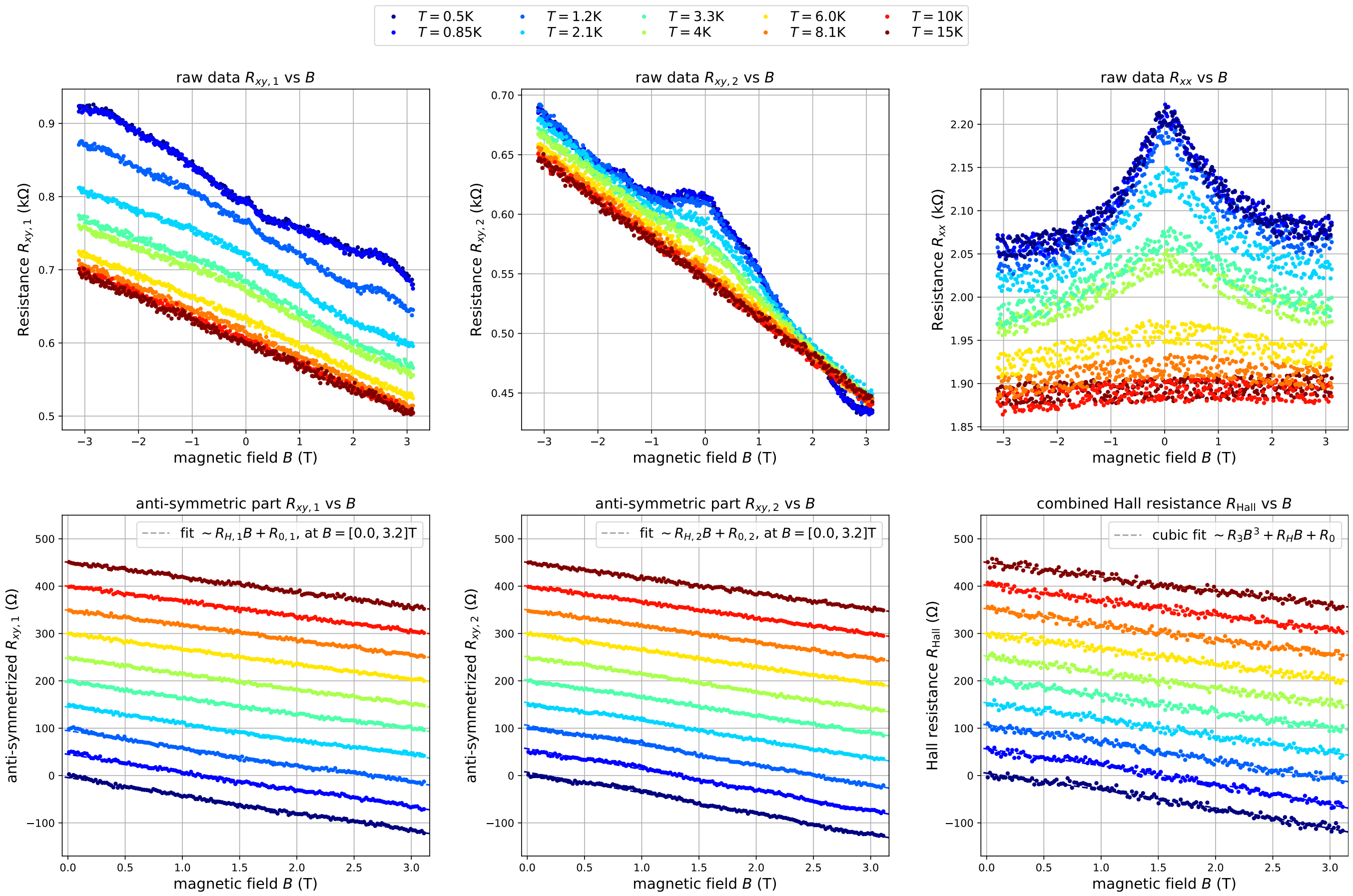

FIG. S8. **Magneto-transport measurements and Hall coefficient fits for device B [backward field sweep, large field range].** Top row: raw magneto-transport data for the transverse resistances $R_{xy,1}$ (left) and $R_{xy,2}$ (center), and the longitudinal resistance R_{xx} (right) measured during a backward sweep of the magnetic field $B = 3.2\,\mathrm{T} \to -3.2\,\mathrm{T}$ at temperatures $T \in [0.5\,\mathrm{K}, 15.0\,\mathrm{K}]$ (see legend) for device B. Bottom row: field-antisymmetric parts of $R_{xy,1}$ (left) and $R_{xy,2}$ (center), with $50\,\Omega$ shifts between consecutive temperatures, and a linear fit (dashed lines) that determines the respective Hall coefficient $R_{H,1/2}$ and a zero-field offset $R_{0,1/2}$. The Hall resistance R_{Hall} (cf. Eq. (2); right plot, with $50\,\Omega$ shifts) is fitted by a similar linear fit or by a linear-plus-cubic polynomial in the full field range (dashed lines). Results of the fits are shown in Fig. S9.

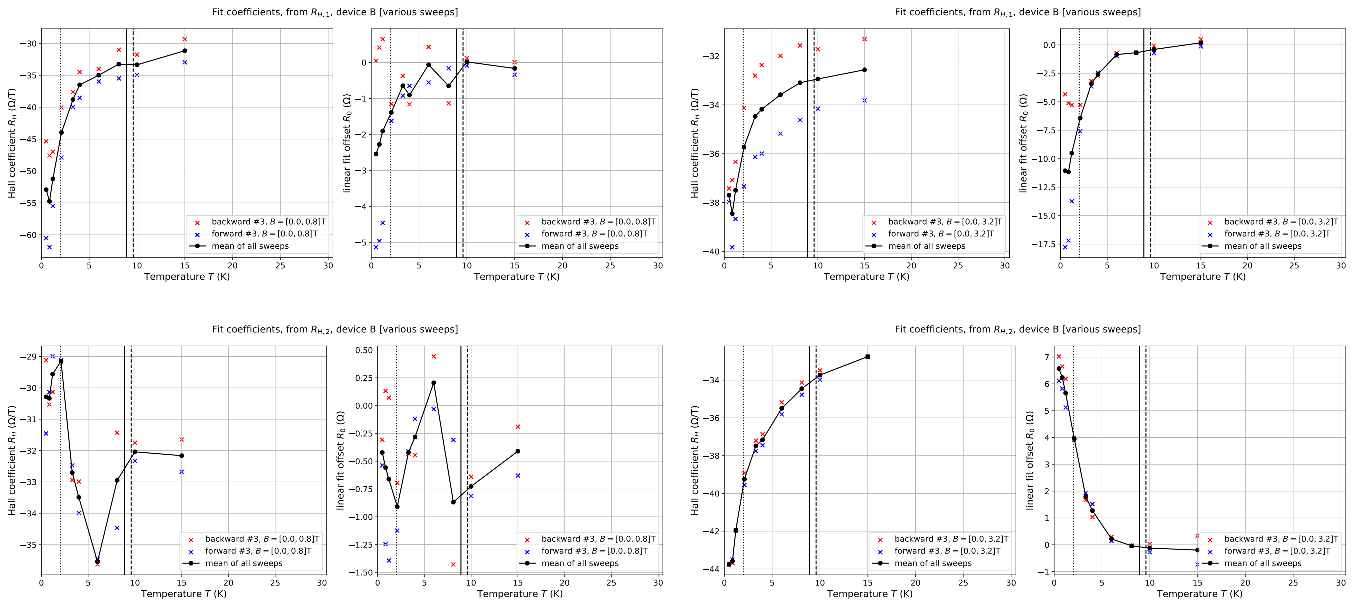

FIG. S9. **Reproducibility of the Hall coefficient R_H and offset R_0 for device B, fitted to different field ranges.** Top left: R_H and R_0 from fits of $R_{xy,1}$ in the window $B = [0.0, 0.8]$T for the large-range field sweeps in Figs. S7 and S8. Black dots and lines indicate the average value, while red/blue symbols indicate the individual backward/forward sweep data. Bottom left: same as top left, but with coefficients from the linear fit to $R_{xy,2}$ instead. We find different but consistent trends as obtained for the smaller field-range sweeps in Figs. S3-S6, which were also fitted in the low-field window $B = [0.0, 0.8]$T. Top and bottom right: same as on the left, but with R_H and R_0 obtained from fits to $R_{xy,1}$ (top) and $R_{xy,2}$ (bottom) in the full field range window $B = [0.0, 3.2]$T. Compared to the low-field slopes (left), we observe that R_H from $R_{xy,2}$ (bottom right) fitted in the large field range also exhibits a gradual down-turn to more negative values at low temperatures. This behavior is qualitatively consistent with the general trend of R_H obtained from $R_{xy,1}$ in large and small field range sweeps (top right, and Fig. S6). The vertical dashed and dotted lines indicate the tunnelling and exchange temperature scales T_t and T_J, cf. Sec. S4 E.

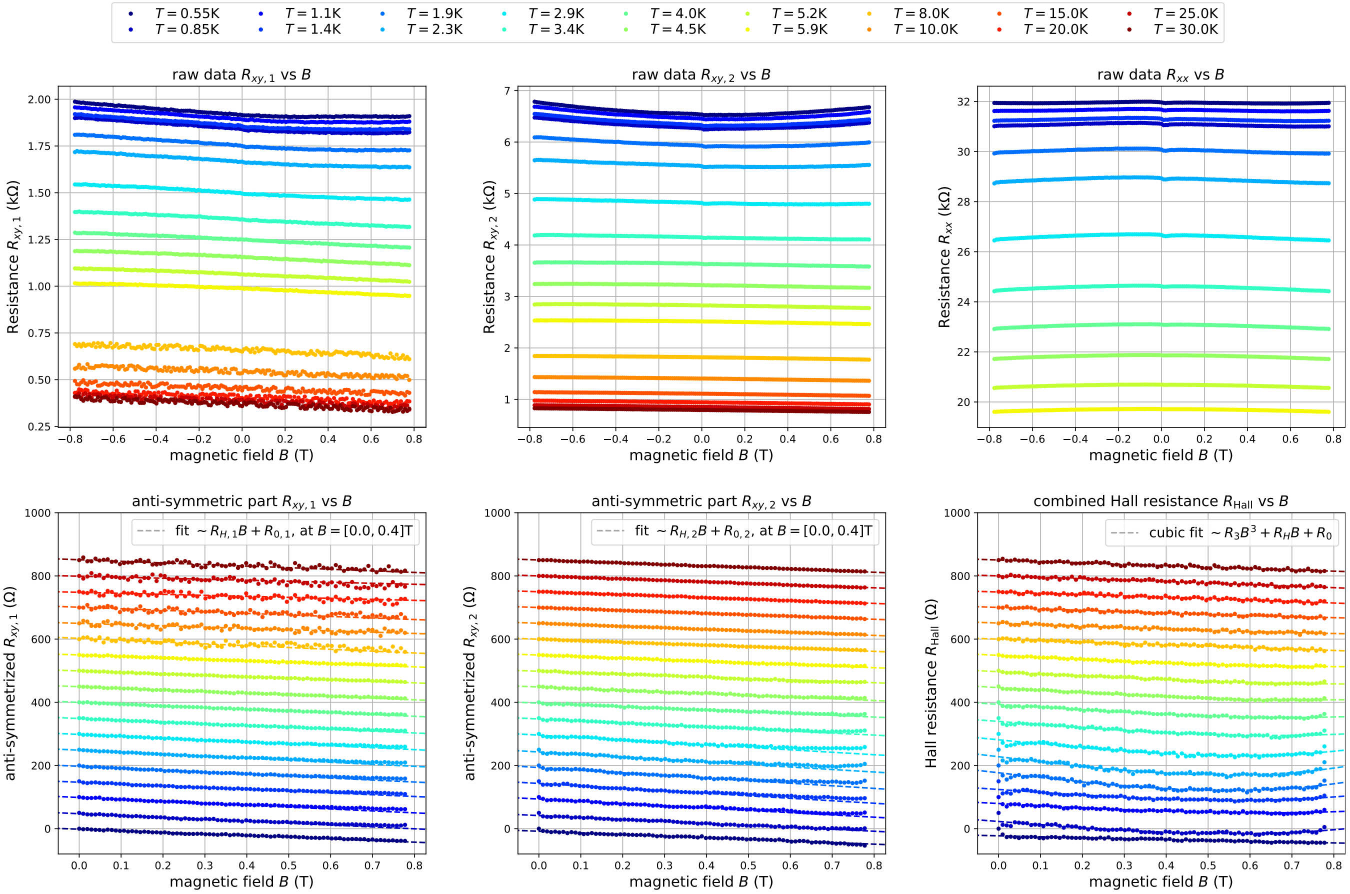

FIG. S10. **Magneto-transport measurements and Hall coefficient fits for device C [forward field sweep].** Top row: raw magneto-transport data for the transverse resistances $R_{xy,1}$ (left) and $R_{xy,2}$ (center), and the longitudinal resistance R_{xx} (right) measured during a forward sweep of the magnetic field $B = -0.8\,\mathrm{T} \rightarrow 0.8\,\mathrm{T}$ at various temperatures $T \in [0.55\,\mathrm{K}, 30.0\,\mathrm{K}]$ (see legend) for device C. Bottom row: field-antisymmetric parts of $R_{xy,1}$ (left) and $R_{xy,2}$ (center), with $50\,\Omega$ shifts between consecutive temperatures, and a linear fit (dashed lines) that determines the respective Hall coefficient $R_{H,1/2}$ and a zero-field offset $R_{0,1/2}$. The Hall resistance R_{Hall} (cf. Eq. (2); right plot, with $50\,\Omega$ shifts) is fitted by a similar linear fit at low fields $B < 0.4\,\mathrm{T}$ or a linear-plus-cubic polynomial in the full field range (dashed lines). Results of the fits are shown in Fig. S12.

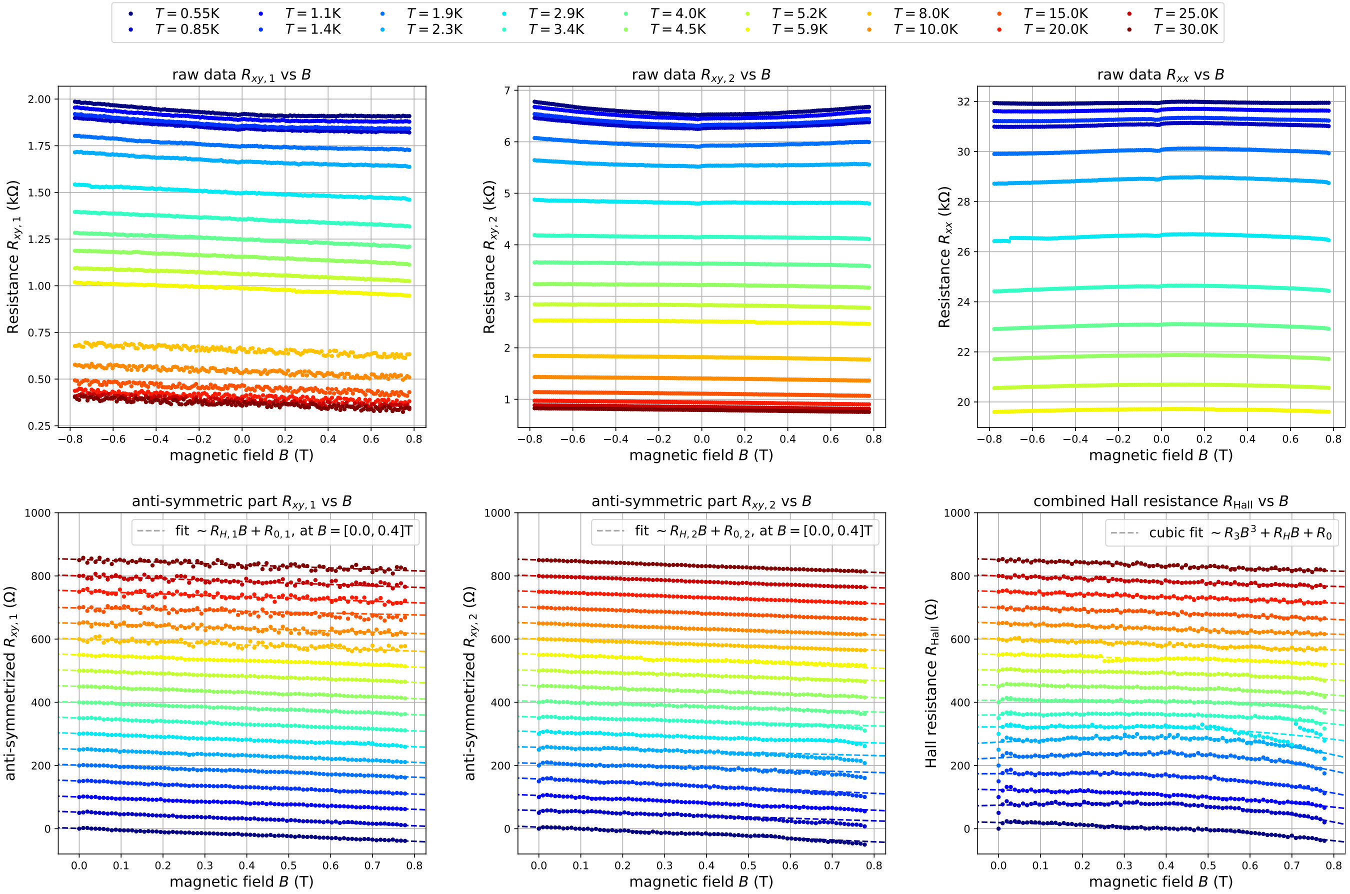

FIG. S11. **Magneto-transport measurements and Hall coefficient fits for device C [backward field sweep].** Top row: raw magneto-transport data for the transverse resistances $R_{xy,1}$ (left) and $R_{xy,2}$ (center), and the longitudinal resistance R_{xx} (right) measured during a backward sweep of the magnetic field $B = 0.8\,\mathrm{T} \rightarrow -0.8\,\mathrm{T}$ at various temperatures $T \in [0.55\,\mathrm{K}, 30.0\,\mathrm{K}]$ (see legend) for device C. Bottom row: field-antisymmetric parts of $R_{xy,1}$ (left) and $R_{xy,2}$ (center), with $50\,\Omega$ shifts between consecutive temperatures, and a linear fit (dashed lines) that determines the respective Hall coefficient $R_{H,1/2}$ and a zero-field offset $R_{0,1/2}$. The Hall resistance R_{Hall} (cf. Eq. (2); right plot, with $50\,\Omega$ shifts) is fitted by a similar linear fit at low fields $B < 0.4\,\mathrm{T}$ or a linear-plus-cubic polynomial in the full field range (dashed lines). Results of the fits are shown in Fig. S12.

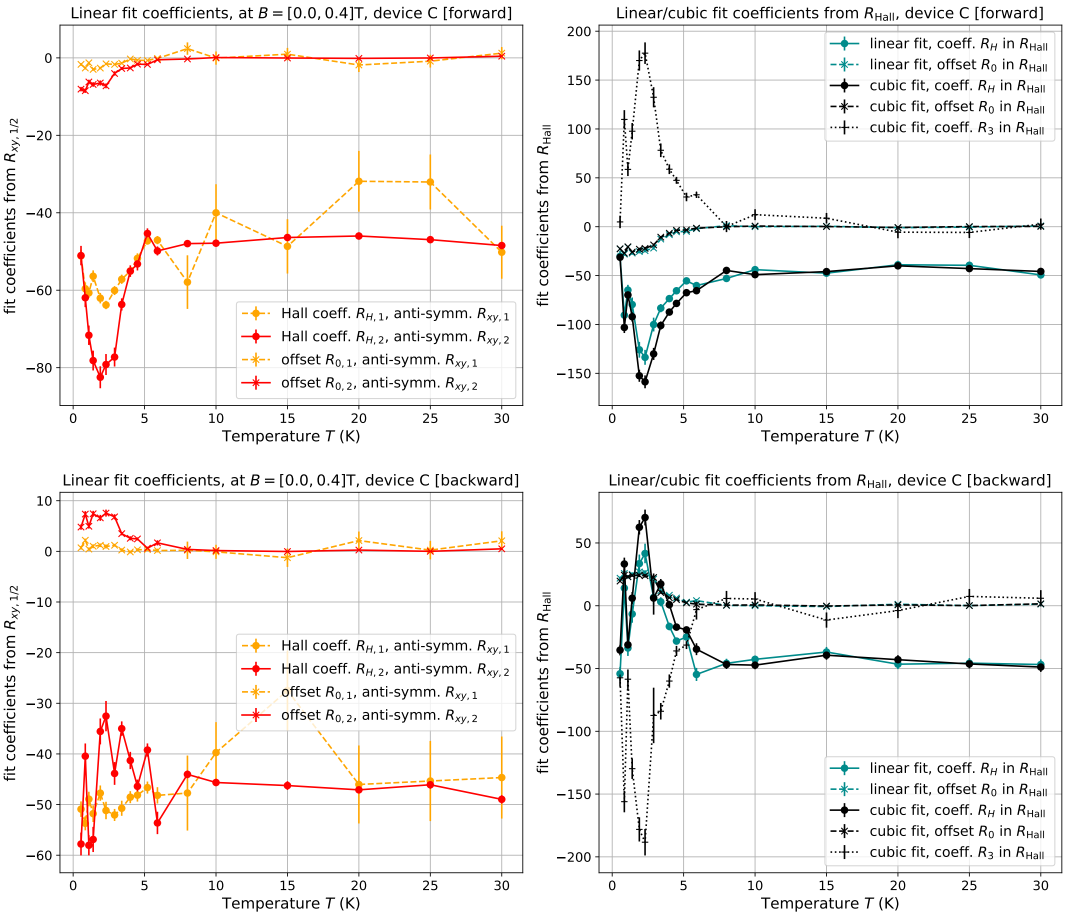

FIG. S12. **Hall coefficient and offset fit parameters for device C [forward and backward field sweeps].** Top row: Hall coefficients and offsets obtained from the forward field sweeps in Fig. S10. Fit values obtained from the anti-symmetrized $R_{xy,1/2}$ (top left) and R_{Hall} (top right) are qualitatively consistent, though $R_{xy,1}$ exhibits stronger variations/noise also visible in the raw data. The Hall coefficient $R_{H,2}$ (from $R_{xy,2}$) and R_H (linear/cubic fits of R_{Hall}) shows a flat temperature dependence above $T \approx 10\,\mathrm{K}$, a strong down-turn to more negative values at $T \lesssim 5\,\mathrm{K}$ that bottoms out at $T \approx 2.5\,\mathrm{K}$, and a turn back up at the lowest temperatures. Similarly, a finite negative offset R_0 develops at low temperatures (in $R_{xy,2}$ and R_{Hall}). Bottom row: Hall coefficients and offsets obtained from the backward field sweeps in Fig. S11. The Hall coefficient $R_{H,2}$ and R_H shows a flat temperature dependence above $T \approx 10\,\mathrm{K}$, consistent with the forward sweep. The low-temperature data however is distinct and exhibits less clear or consistent trends between $R_{H,2}$ and R_H. Opposite to the forward sweep (top right), R_H shows an upward spike to positive values at low temperatures $T \lesssim 10\,\mathrm{K}$ in the backward sweep (bottom right). A positive offset R_0 develops at low temperatures, in $R_{xy,2}$ and R_{Hall}, again opposite to the forward sweep. Differences between forward and backward sweeps may be associated with magnetic hysteresis, and appear much weaker in the less strongly interacting devices A and B or at elevated temperatures $T \gtrsim T_c$ above the electron coherence scale $T_c \approx 17\,\mathrm{K}$ in device C. For further analysis, see Fig. S14.

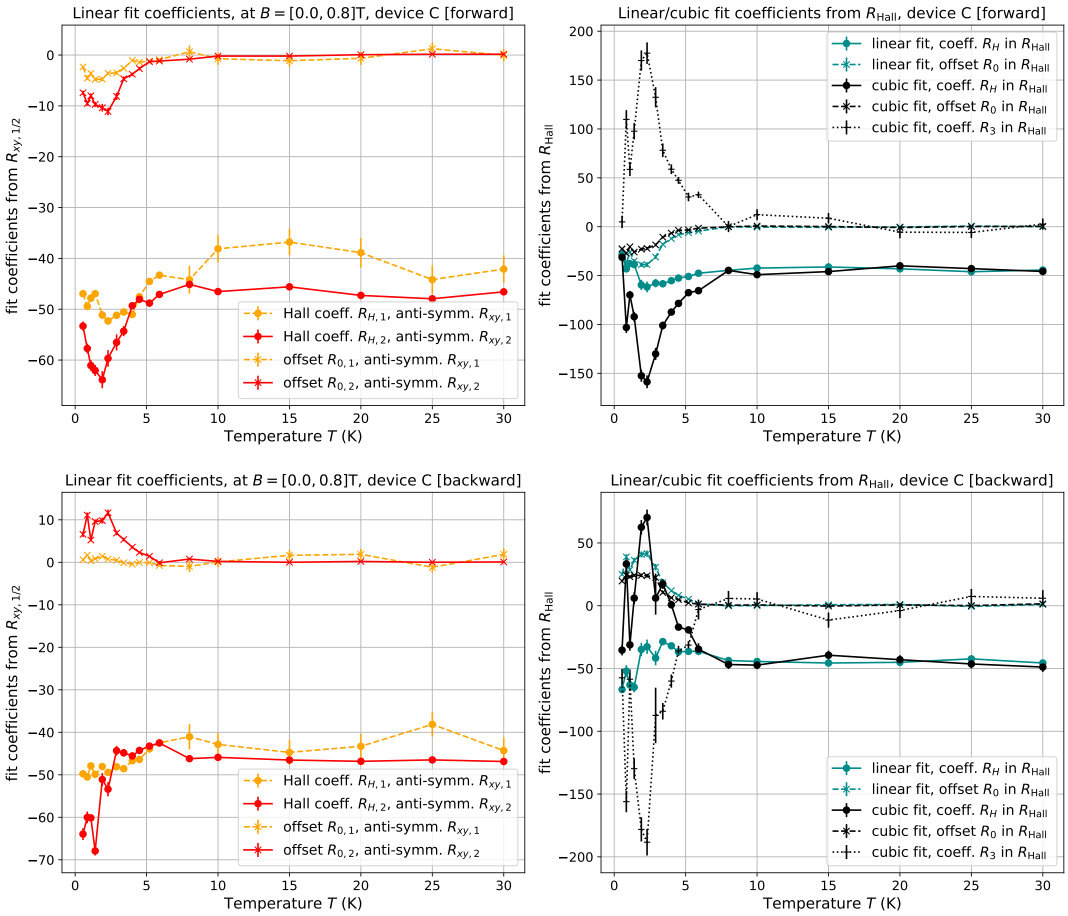

FIG. S13. **Hall coefficient and offset fit parameters for device C [forward and backward field sweeps, full range fit].** Same as Fig. S12, but with linear fits of R_H and R_0 obtained from $R_{xy,1}$, $R_{xy,2}$, and R_{Hall} in the full available field range $B = [0.0, 0.8]$T in Figs. S10 and S11. The values for the Hall coefficient R_H at elevated temperatures $T \sim T_c \approx 17\,$K quantitatively agree with the ones obtained from fits in the low-field range $B = [0.0, 0.4]$T, cf. Fig. S12. The low-temperature behavior of R_H, discussed in Fig. S12, is recovered on a qualitative level also for the fit based on the larger magnetic field range, particularly for the forward magnetic field sweep (top row).

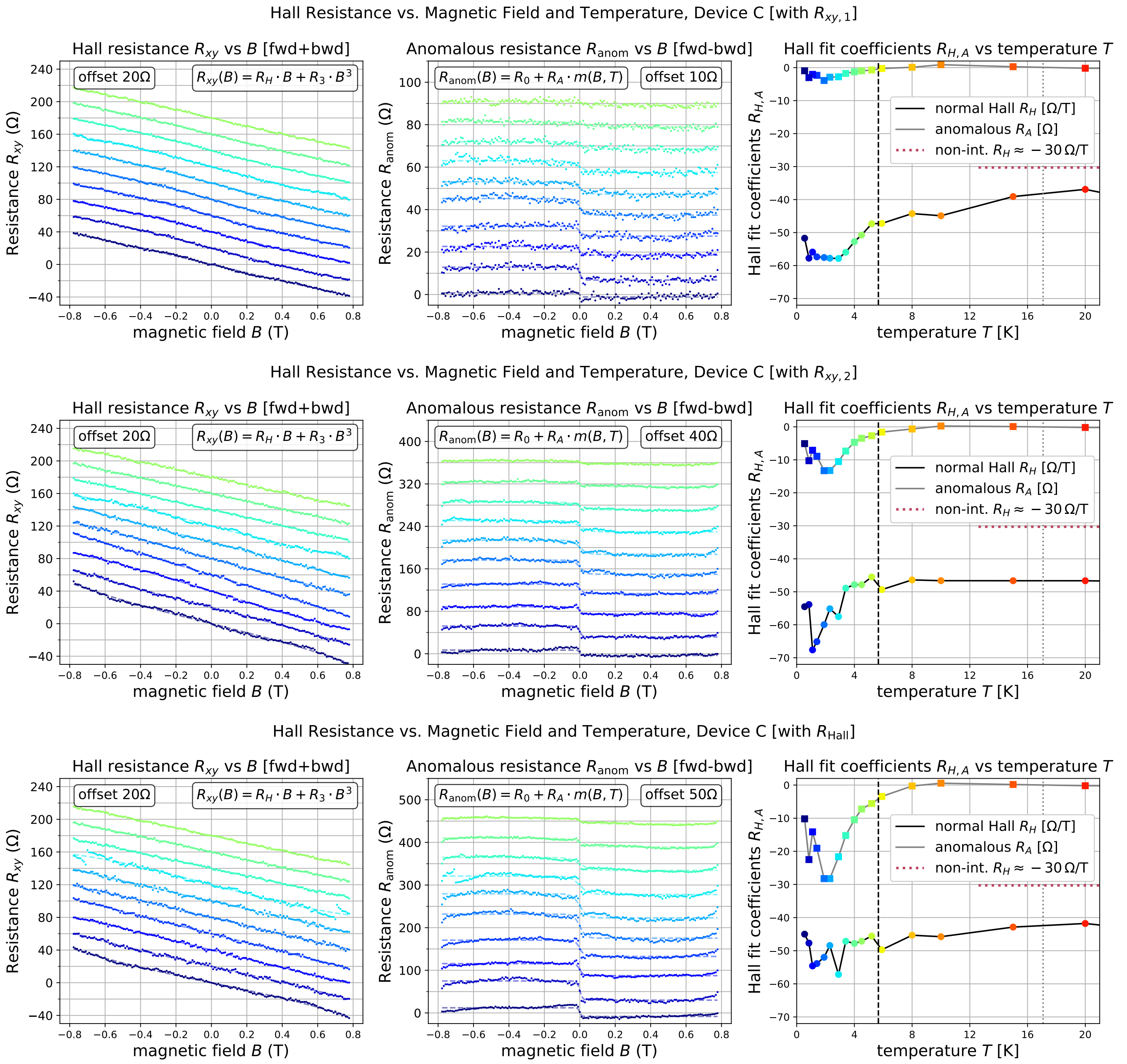

FIG. S14. **Signatures of magneto-hysteresis from Hall coefficient and anomalous resistance fits, for device C.** The normal part of the Hall resistance (left panel) is obtained from the average, while the anomalous resistance (centre panel) refers to the difference of forward and backward magnetic field sweeps in Figs. S10 and S11, respectively. Top row: obtained from the transverse resistance $R_{xy,1}$ with magnetic field $B \in [-0.8\,\mathrm{T}, 0.8\,\mathrm{T}]$ at temperatures $T \in [0.55\,\mathrm{K}, 30\,\mathrm{K}]$ (color-coded, right panel). Centre row: from the transverse resistance $R_{xy,2}$. Bottom row: from the total Hall resistance R_{Hall} that includes odd-in-field contributions of the longitudinal resistances, see Eq. (2). In left and centre panels for clarity we only plot a few of the raw data curves, with offsets as indicated. The normal Hall resistances are fit to a linear-plus-cubic polynomial (left panel, cf. Figs. S12 and S13), and the anomalous resistances are fit to a term $\sim R_A \cdot m$ with magnetization $m(B,T)$ (centre panel, dashed lines) with $m(B,T) = \tanh\left(\frac{g_{\mathrm{eff}}\mu_B B}{k_B T}\right)$ and a large $g_{\mathrm{eff}} \gg g_0 = 2$. The resulting Hall coefficient R_H and anomalous resistance prefactor R_A for all temperatures are shown in the right-hand plots, and are in qualitative agreement for fits to the either of the transverse or full Hall resistances. The fitted Hall coefficients $R_H(T)$ agree with the ones obtained from individual forward or backward magnetic field sweeps in Figs. S12 and S13, when the appropriate averages are taken.

S3. MODELLING OF THE HUBBARD PARAMETERS AND DISORDER

A. On-site interaction U

The on-site interaction energy U in the Fermi-Hubbard Hamiltonian encodes the Coulomb repulsion between electrons occupying the same quantum dot. We can engineer the value of U by controlling the area of the quantum dots, in which U decreases as the quantum dot area increases. In Figure S15a we plot U as a function of quantum dot area A, calculated by solving the electrostatics of a single quantum dot using a finite element solver (COMSOL) [14, 15]. The interaction energy can be expressed as $U = e^2/2C$ where e is the elementary electron charge and C is the self-capacitance of the quantum dot obtained from the diagonal elements of the capacitance matrix [15–18]. The range of quantum dot areas used in this work ($9\,\mathrm{nm}^2$ to $64\,\mathrm{nm}^2$) was chosen to achieve suitable, large on-site interaction energies (15-40 meV) while minimising disorder in U from small variations in the quantum dot size (see Section S3 D for estimates of the disorder strength). We note that the data in Fig. S15a follows $U \sim 1/A^\alpha$ with $\alpha \approx 0.38$, which roughly matches the Coulomb energy $U_\square \sim 1/\sqrt{A}$ in a uniformly charged square or an electron jellium model [19, 20]. For device E, with quantum dot size $\sim 9\mathrm{nm}^2$, in our experiments we found that the interaction energy $U_{\mathrm{COMSOL}} \approx 32.9\,\mathrm{meV}$ represents an underestimate. For such small nanostructures, the sharp electron confinement in donor quantum dots necessitates a revision of the interactions calculation on the basis of Hartree-Fock self-consistent field simulations [21, 22]. We find an on-site interaction $U_{\mathrm{SCF}} \approx 48.0\,\mathrm{meV} \gg U_{\mathrm{COMSOL}} \approx 32.9\,\mathrm{meV}$ [21], enhanced by a factor $U_{\mathrm{SCF}}/U_{\mathrm{COMSOL}} \approx 1.46$ compared to the larger quantum dot devices in which geometric capacitance contributions outweigh the quantum confinement effects. For device E, throughout this work we hence use the larger, rescaled interaction values U_{SCF}, V_{SCF}, $\cdots$ based on the more accurate HF-SCF simulations. While this only provides a rough estimate for the enhanced long-range interactions $V_{\mathrm{SCF}}, \cdots$, the direct HF-SCF simulation of a large-scale (9×9) quantum dot array needed to capture long-range interaction and screening effects is infeasible. In Table S1 we quote both HF-SCF rescaled interaction energies and the COMSOL simulation results (in brackets).

B. Inter-site interactions V, V', and V''

The inter-site interaction energies V, V', and V'' in the extended Fermi-Hubbard Hamiltonian encode the Coulomb repulsion between electrons occupying distinct nearby quantum dots. To model and extract their values, we simulate a 9×9 grid of quantum dots (at this size of the dot array, finite-size and boundary effects are negligible) and calculate its capacitance matrix with a finite element solver (COMSOL) [14, 15]. Using the constant interaction approximation, inter-site interactions are expressed as $V_{ij} = e^2/C_m \cdot 1/(C_i C_j / C_m^2 - 1)$ where C_m is the mutual capacitance between the central dot and a neighbouring dot, and C_i and C_j are the total capacitance of the central dot and the neighbouring dot, respectively [15–18]. In Figure S15b, we plot the nearest-, next-nearest, and next-next-nearest neighbour inter-site interactions V, V', and V'' as a function of the separation between the quantum dots for three different dot areas. As the inter-dot distance a increases, the inter-site interactions tend to become weaker. They are largest for the smallest-area dots, which also exhibit the strongest dependence of V, V', and V'' on the separation a. Note that inter-site interactions for next-nearest neighbours V' are typically 50% to 70% of the nearest-neighbour value V.

C. Electron hopping amplitudes t and t'

The strength of the nearest- and next-nearest neighbour electron hopping amplitudes t and t' are determined from atomistic tight-binding simulations of the donor island array using molecular orbital theory [22]. Primarily dependent on the inter-dot separation $a \simeq 7\,\mathrm{nm} - 15\,\mathrm{nm}$, from theory simulations, we find typical next-neighbour hopping energies for the quantum dot arrays fabricated in this work to fall between $t \approx 0.05\,\mathrm{meV}{-}2\,\mathrm{meV}$, see Figure S15c. These broadly agree with hopping strengths quoted in other earlier theory simulations [23–26] and for similar devices with fewer, smaller quantum dots [15]. Values for the next-nearest neighbour electron hopping t' (between diagonally separated quantum dots) are $\sim 3\%$ of the next-neighbour value t in the donor quantum dot square lattice at any fixed inter-dot distance a, see Figure S15c.

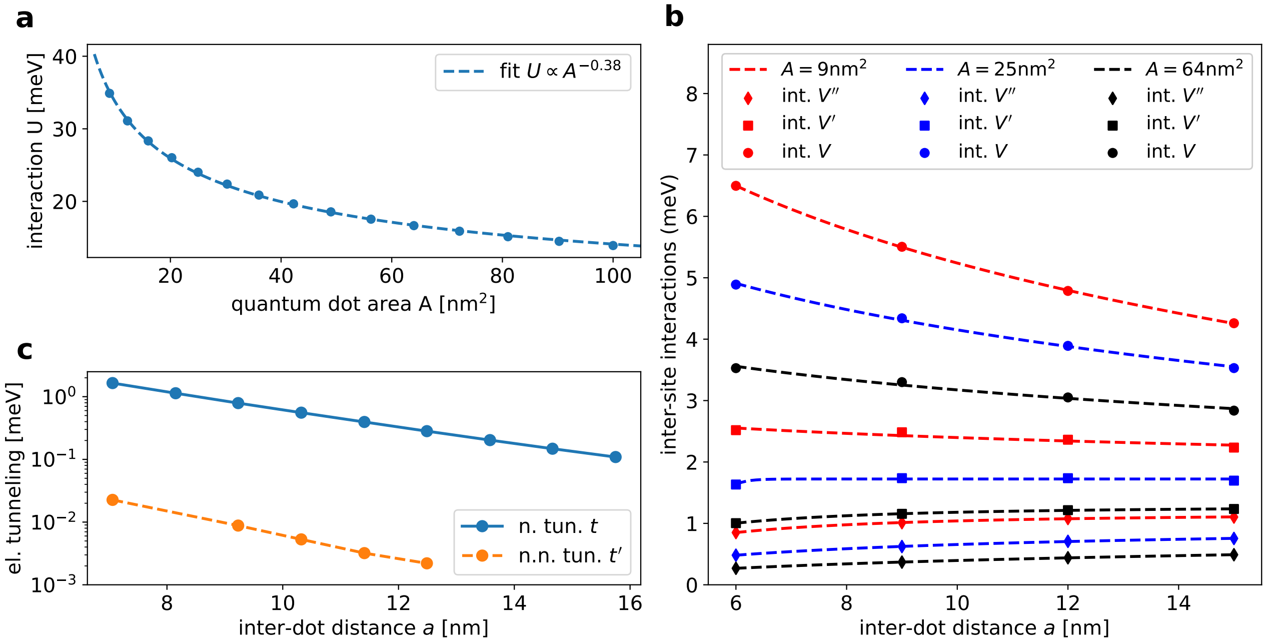

FIG. S15. **Theoretical calculations of the Hubbard parameters for our atom-based quantum dots. a.** The on-site interaction, U, of a single quantum dot as a function of the dot area, A, simulated using the COMSOL finite-element solver. **b.** Various inter-site interactions as a function of the inter-dot distance a. Here the values V, V', and V'', refer to inter-site interactions between neighbouring quantum dots (separated by a), next-neighbour dots (separated diagonally, by $\sqrt{2}a$), and next-next neighbour dots (separated by $2a$) on the square lattice. Notice that longer-range interactions decay slowly or might even rise as the inter-dot distance a is increased, the total interaction cost of an electron with all of its neighbors however always reduces as the inter-dot distance or dot area grow. **c.** The electron hopping amplitudes t and t' as a function of the inter-dot distance a for a quantum dot of size $A = 25\,\text{nm}^2$. The solid and dashed line (blue and orange symbols) represent the hopping amplitude between neighbouring quantum dots (separated by a) and next-neighbour dots (separated by $\sqrt{2}a$), respectively.

D. Disorder effects

The disorder in the Hubbard parameters $t, U, V, \cdots$ in our quantum dot arrays can be estimated based on variation in the inter-dot distance a and dot area A. Using scanning tunnelling microscope (STM) images we can compute the mean and standard deviation for both a and A in each array, using a sample of 30 quantum dots spread throughout the array. The obtained values are noted in Table S1. We then use the relationship between the Hubbard parameters and the quantum dot area and separation modelled in Sections S3 A-S3 C and Fig. S15, to determine the mean and standard deviation of U, V, and t, via the so-called variance formula

$$\sigma_U^2 = \left(\frac{\partial U}{\partial A}\right)^2 \sigma_A^2 + \left(\frac{\partial U}{\partial a}\right)^2 \sigma_a^2 \,,$$

with equivalent formulas for the other Hubbard parameters. The results for the variances are quoted in Table S1. Taking the standard deviation of the Hubbard parameters as an estimate of the disorder, we observe that the level of disorder in our QD arrays is on the order of $\sigma_t,\ \sigma_U,\ \sigma_V,\ \sigma_{V'} \sim 1\,\text{meV}$ or less.

We note that disorder due to varying local chemical potentials μ_i of the quantum dots in the array is not accounted for at present. Compared to gate-defined quantum dots (or other platforms), our atom-based quantum dots each host many electrons, most of which are tightly bound and do not partake in transport (in the more insulating arrays). Yet these ‘core electrons’ of the artificial atom lattice contribute to electric screening and reduce the effect of electrostatic disorder on the probed ‘valence electrons’ [27, 28]. For quantum dots of the size $A \sim 9\,\text{nm}^2$, $25\,\text{nm}^2$, and $62\,\text{nm}^2$, we expect an incorporation of between 15-22, 42-62, and 105-155 donors, with an equivalent delta-layer doping density of 1.7-2.5 phosphorus donors per nm^2 (see Refs. [6, 29, 30] and our reference delta-layer device in Sec. S2 and Fig. S1). The average number of incorporated donors per dot lies between these values, with a narrower distribution around the mean for the quantum dots in a given device. Due to the resulting high electron density ($\sim$ 0.1-1.0 $\times\ 10^{14}/\text{cm}^2$) in the quantum dot array, external electrostatic disorder within the silicon, interfaces, or the top gate is unlikely to

TABLE S1. **Inter-dot distance a, quantum dot area A, and the corresponding Hubbard parameters t, U, V, and V' for the six quantum dot arrays considered in this work.** The mean and standard deviation (σ) for the inter-dot separation a and quantum dot area A were estimated using STM images of 30 quantum dots spread throughout each array. The mean values of the Hubbard parameters were obtained from a direct COMSOL simulation of the devices A-F with geometry a and A, except for device E where the COMSOL interaction energies (in brackets) were rescaled according to a more accurate but costly Hartree-Fock SCF simulation, see Sec. S3 A for details. The standard deviations σ were estimated using the approximate relation of the Hubbard parameters to the array geometry a and A, extrapolated from the modelling data in Sec. S3 and Fig. S15, for details see Sec. S3 D.

Device	Array Parameter	Mean	σ	Hubbard Parameter	Mean (meV)	σ (meV)
A	a	7.24 nm	0.56 nm	t	1.54	0.28
	A	22.15 nm^2	2.27 nm^2	U	20.90	1.01
				V	4.82	0.19
				V'	1.79	0.08
B	a	9.14 nm	0.52 nm	t	0.83	0.14
	A	27.00 nm^2	2.93 nm^2	U	19.96	0.97
				V	4.23	0.14
				V'	1.69	0.07
C	a	10.75 nm	0.50 nm	t	0.49	0.08
	A	25.83 nm^2	3.30 nm^2	U	20.81	1.15
				V	4.03	0.13
				V'	1.72	0.07
D	a	15.50 nm	0.59 nm	t	0.10	0.02
	A	28.25 nm^2	1.74 nm^2	U	20.87	0.54
				V	3.40	0.08
				V'	1.63	0.03
E	a	15.06 nm	0.55 nm	t	0.12	0.02
	A	8.86 nm^2	0.86 nm^2	U	47.96 (32.91)	1.89 (1.30)
				V	6.21 (4.26)	0.17 (0.12)
				V'	3.26 (2.24)	0.10 (0.07)
F	a	17.07 nm	1.38 nm	t	0.06	0.03
	A	62.15 nm^2	5.21 nm^2	U	14.91	0.55
				V	2.72	0.11
				V'	1.24	0.04

strongly affect the physics of our devices [27]. The main concern then is the disorder in the number and position of dopants in the array itself, where again due to the large number of electrons per quantum dot we expect a highly efficient screening of potential disorder to take place [27, 28]. A detailed analysis and theoretical modelling of the effect of dopant number and positional disorder in large arrays of donor quantum dots is the subject of ongoing research. The complexity of the underlying large-scale numerical simulations and necessary statistical analysis with regards to different types of disorders and their effect on the physics of the devices exceeds the scope of the present work. As a preliminary result, we estimate that the on-site chemical potential energy variations fall in the range of $\delta\mu \sim$1-few meV (of similar order as the quantum dot level spacing δ). This range of values is obtained from self-consistent Schroedinger-Poisson calculations of the electron bound states [22] in a single disordered quantum dot without long-range screening or a full embedding into a large quantum dot array, and as such can be taken as an approximate upper bound. For a detailed discussion of chemical potential disorder in analogue quantum simulators built from different types of quantum dots, we refer to Ref. [27].

S4. CHARGE TRANSPORT THEORY AND DATA ANALYSIS

A. High-temperature transport in granular metals

At high temperatures, charge transport in the atom-based quantum dot arrays can be understood by the theory of granular metals [8, 10, 11, 31]. Here, individual orbital states in the quantum dots and the electron phase coherence in tunnelling through the array are largely unimportant. Instead we focus on the characteristic energy scales of the system, i.e., the typical quantum dot level spacing δ, on-site interaction U, inter-dot tunnelling t, and temperature T.

The conductance $\sigma_0 = \frac{2e^2}{h} g_T$ of the devices at a high temperature $T \approx 50\,\mathrm{K}$ is determined by the inter-dot

conductance $g_T = (2\pi\nu t)^2$, which depends on the tunnelling t and density of states in the quantum dots $\nu = \delta^{-1}$. After measuring σ_0 for each array A-F (taking the average for $T \geq 40\,\mathrm{K}$), with values t determined from our theory modelling (Supplementary S2), we thus are able to estimate the quantum dot level spacings $\delta = 2\pi t/\sqrt{g_T}$. We find

device	A	B	C	D	E	F
g_T	4.538	2.906	1.045	0.125	0.0039	0.413
t [meV]	1.54	0.83	0.49	0.10	0.12	0.06
δ [meV]	4.55	3.05	3.00	1.83	12.05	0.61

(3)

The inter-dot conductances for the insulating devices C-F are revised in Sec. S4 B, and the dot level spacings δ are plotted in Fig. 4e of the main text. We find a smaller level spacing δ the larger the quantum dots, consistent with the idea that large dots host more donor atoms and become metallic as $\sqrt{A} \gg a_{\mathrm{Si}} = 0.543\mathrm{nm}$. Whether a quantum dot array shows metallic or insulating behaviour at low temperatures, i.e. the metal-insulator transition, is set by whether g_T is larger or smaller than a critical value $g_c = \frac{1}{2}\log(U/\delta)$ [11, 32]. For Samples A-D that were fabricated to have similar dot parameters δ and U, we find the critical conductance $g_c \approx 1.11$ ($\sigma_c = \frac{2e^2}{h} g_c$) which is indicated in Fig. 3b of the main text. Going forward we are able to fabricate devices with various quantum dot and lattice geometries, see Figs. 1 and 2 of the main text, that are targeted to fall close to or far from the predicted metal-insulator transition.

B. Electron co-tunnelling and thermal activation

A transition from inelastic to elastic co-tunnelling of electrons in the quantum dot arrays, starting from the high-temperature granular metal limit, is set by the cross-over temperature $k_B T_c \approx 0.2\sqrt{\delta U}$ [11, 12, 33]. For temperatures $T > T_c$, electrons in the insulating devices C-F move across the dots via inelastic co-tunnelling processes, giving rise to a thermal activation of conduction described by an Efros-Shklovskii (ES) law [11, 31, 34]

$$\sigma_{\mathrm{ES}}(T) = \sigma_0 \exp(-\sqrt{T_0/T}). \tag{4}$$

The data in Fig. 4 of the main text matches this prediction, where the dashed vertical lines indicate $T_c \approx 9.0\mathrm{K}, 29.0\mathrm{K}$, and 5.5K for Samples D, E, and F, obtained from the respective scales δ and U. The activation temperature T_0 is larger for the more strongly interacting arrays, with the fits of $\sigma_{\mathrm{ES}}(T > T_c)$ in Fig. 4d of the main text yielding the values T_0^{in}. At low temperatures theory again predicts a ES law but with a distinct activation scale T_0^{el}. In both regimes T_0 is compared to the on-site interaction strength $k_B T_0 \simeq U/\xi_0$, where ξ_0 is interpreted as the localization length of electrons in units of the dot lattice spacing a [11, 12, 33]. For the (near) insulating devices C-F, we find

device	g_T[ES]	δ_{ES}[meV]	T_c[K]	U[meV]	T_0^{in}[K]	ξ_0^{in}	T_0^{el}[K]	ξ_0^{el}	$\xi_{\mathrm{th}}^{\mathrm{el}}$
C	1.387	2.60	17.1	20.81	3.41	70.9	–	–	0.38
D	0.812	0.72	8.98	20.87	162	1.49	–	–	0.31
E	0.053	3.25	29.0	47.96	318	1.75	3325	0.17	0.25
F	1.091	0.37	5.47	14.91	44.3	3.90	519	0.33	0.29

(5)

Compared to the Table Eq. (3), a fit to the ES law yields a larger value for the inter-dot conductance $g_T = \frac{h}{2e^2}\sigma_0$. This is unsurprising, since for the insulating devices a full thermal activation is not achieved in the accessible temperature range $T \lesssim 50\,\mathrm{K}$, see Fig. 3 and 4d of the main text. Following Sec. S4 A, a larger estimate g_T leads to a reduced dot level spacing δ_{ES}. In Fig. 4e of the main text, we then show the quantum dot level spacing δ as obtained in Sec. S4 A for the metallic devices A and B, and the above result of the ES fit for the insulating devices C, D, E, and F.

Existing theory for granular metals [11, 12, 33] demands that the localization lengths are small, $\xi_0 \ll 1$, such that electrons are confined to within a single quantum dot. While at low temperatures $T < T_c$ this holds for samples E and F, in the inelastic co-tunnelling limit at elevated temperature all devices host fairly delocalized electrons. We attribute this to the large inter-dot conductances g_T, whereas theoretical analysis works by treating $g_T \ll 1$ as a small parameter [12, 33]. This assumption holds only approximately for devices D and E, and is clearly violated for device C that we found to be close to the metal-insulator transition, cf. Sec. S4 A and Fig. 3 of the main text. The delocalized electrons in our devices are able to more efficiently resist freezing out as temperature is lowered, as observed in Fig. 4d of the main text. Taking the theory prediction $\xi_{\mathrm{th}}^{\mathrm{el}} = 2/\ln\left(\frac{2\pi^2 U}{c g_T \delta}\right)$ [11, 12, 33] with constant $c \approx 1$ and inserting our device parameters yields $\xi_{\mathrm{th}}^{\mathrm{el}} \approx 0.25,\ 0.29$ for samples E and F, in reasonable agreement with the experimentally determined ξ_0^{el}. Last, we do not seem to observe an elastic co-tunnelling regime at low temperatures $T < T_c$ in device D. This might be due to charge noise or other detrimental effects that reduce the electron phase coherence in this device. From the analysis and results presented here and in Fig. 4 and discussion of the main text,

we conclude that temperatures $T < \min\left[T_c, \frac{\delta}{k_B}\right]$ hold most promise for coherent electron many-body physics in our atom-based quantum dot arrays, informing additional experiments and future device design.

C. Electron excitations and the charge transport gap

A complementary tool to investigate electron interaction effects in our quantum dot arrays are measurements of the charge transport gap Δ_c, from voltage-bias spectroscopy of the longitudinal voltage in the Hall bar devices. The data and analysis presented here focuses on the insulating devices D-F, see Fig. 4 of the main text. We then need to distinguish between two distinct excitation gaps, for single-particle excitations (the electron/charge addition energy) and two-particle electron-hole excitations (the cost to create an electron-hole charge dipole on a pair of adjacent sites). The Mott-Hubbard charge gap for single-electron (single-charge) excitations in the quantum dot array is given by

$$\Delta_c^{\rm th} = \frac{1}{2}U + 4V + 4V' + 4V_{2,0} + 8V_{2,1} + 4V_{2,2} + \cdots = V_{\rm c}^{\rm tot} - \frac{1}{2}U \ , \tag{6}$$

with the on-site interaction U, the inter-site interactions V and V' (next and next-nearest neighbour), and longer-range interactions $V_{x,y}$ between sites that are removed by (x, y) steps in the quantum dot lattice. The last equality notes the single-excitation Mott gap in terms of the on-site interaction U and the total Coulomb interaction $V_{\rm c}^{\rm tot}$, which is the integrated Coulomb potential of a single electron charge at the origin with respect to electrons on the same and all surrounding sites, $V_{\rm c}^{\rm tot} = \sum_{\vec{r}\in \text{ lattice}} V_{\rm c}(r)$. This expression is a general mean-field result for two-body Coulomb interactions and the addition energy cost of an electron in a 'granular' array of interacting electronic sites, and not necessarily limited to an ordered (Fermi-Hubbard) quantum dot lattice system.

The Mott-Coulomb gap for electron-hole excitations in the quantum dot array, i.e. the cost of creating a charge dipole between neighbouring sites by moving any electron from its site to a neighbouring one, in contrast reads [11, 12]

$$\Delta_{\rm eh}^{\rm th} = U - c g_T E_{\rm eh}, \qquad \text{with} \ \ c = \frac{4}{\pi^2}\ln(2) \approx 0.281, \quad E_{\rm eh} = 2U - V, \tag{7}$$

with the bare excitation energy cost $E_{\rm eh}$ for creating an electron-hole dipole between neighbouring sites; $g_T = (2\pi t/\delta)^2$ is the dimensionless inter-dot conductance, cf. Sec. S4 A. The Mott-Coulomb gap is suppressed for large inter-dot conductances g_T, where the above formula is valid in the co-tunnelling regime $g_T \ll \frac{\pi}{2} \simeq 1.57$, which holds for arrays D-F. In the opposite regime of large g_T, the Coulomb gap is suppressed exponentially as $\Delta_{\rm eh}(g_T \gg 1) \sim U e^{-4\pi g_T}$ down to a value $\sim g_c\delta$ with the dot level spacing δ and critical conductance g_c, cf. Sec. S4 A and Refs. [11, 12].

The interaction energy scales U and V, V', $\cdots$ for all devices are predicted from electrostatics (and HF-SCF) simulation of the dot arrays, cf. Sec. S3 and Table S1. The inter-dot conductances g_T for each array are estimated from measurements of the respective high-temperature conductances in Fig. 3b and Fig. 4d of the main text. We then find the Mott-Hubbard and Mott-Coulomb charge transport gaps for the three insulating arrays D, E, and F, as

device	g_T	U [meV]	V [meV]	V' [meV]	$V_{2,0}$ [meV]	$V_{2,1}$ [meV]	$V_{2,2}$ [meV]	$\Delta_c^{\rm th}$ [meV]	$\Delta_{\rm eh}^{\rm th}$ [meV]
C	1.387	20.81	4.03	1.73	0.67	0.51	0.28	41.3	6.16
D	0.812	20.87	3.40	1.63	0.72	0.56	0.32	39.2	12.1
E	0.053	47.96	6.21	3.26	1.62	1.29	0.79	81.8	46.6
F	1.091	14.91	2.72	1.24	0.52	0.39	0.21	29.3	6.60

(8)

Due to the relatively weak screening of interactions in our quantum dot arrays, we find that the predicted Coulomb gaps $\Delta_{\rm eh}^{\rm th}$ are smaller than the Mott-Hubbard excitation gaps $\Delta_c^{\rm th}$. In voltage-bias spectroscopy experiments, discussed in Fig. 4 of the main text, we therefore should expect to observe signatures of both the Mott-Hubbard and Coulomb gap mechanisms. This is in contrast to other Fermi-Hubbard simulators in which long-range interactions are strongly suppressed. Further, the transport measurements naturally probe single-particle excitations due to electron hopping into/out of the system from the lead contacts in combination with the bulk transport due to particle-hole excitations.

The voltage-bias spectroscopy (VBS) measurements at low temperature (Fig. 4b of the main text) and the extended VBS thermal activation data in Fig. S16 show clear evidence of charge transport gaps in all insulating devices D-F. For device C, due to the close proximity to the metal-insulator transition, it is difficult to resolve a charge transport gap. We now offer a tentative discussion of the VBS thermal activation data in Fig. S16, though a full understanding of the different charge-transport regimes in our atom-based quantum dot arrays may require further study and devices. To this end, note the drastically different inter-dot conductances g_T, varying by two orders of magnitude in devices D-F. Further for the devices studied by us both geometric capacitance and quantum confinement/capacitance effects in

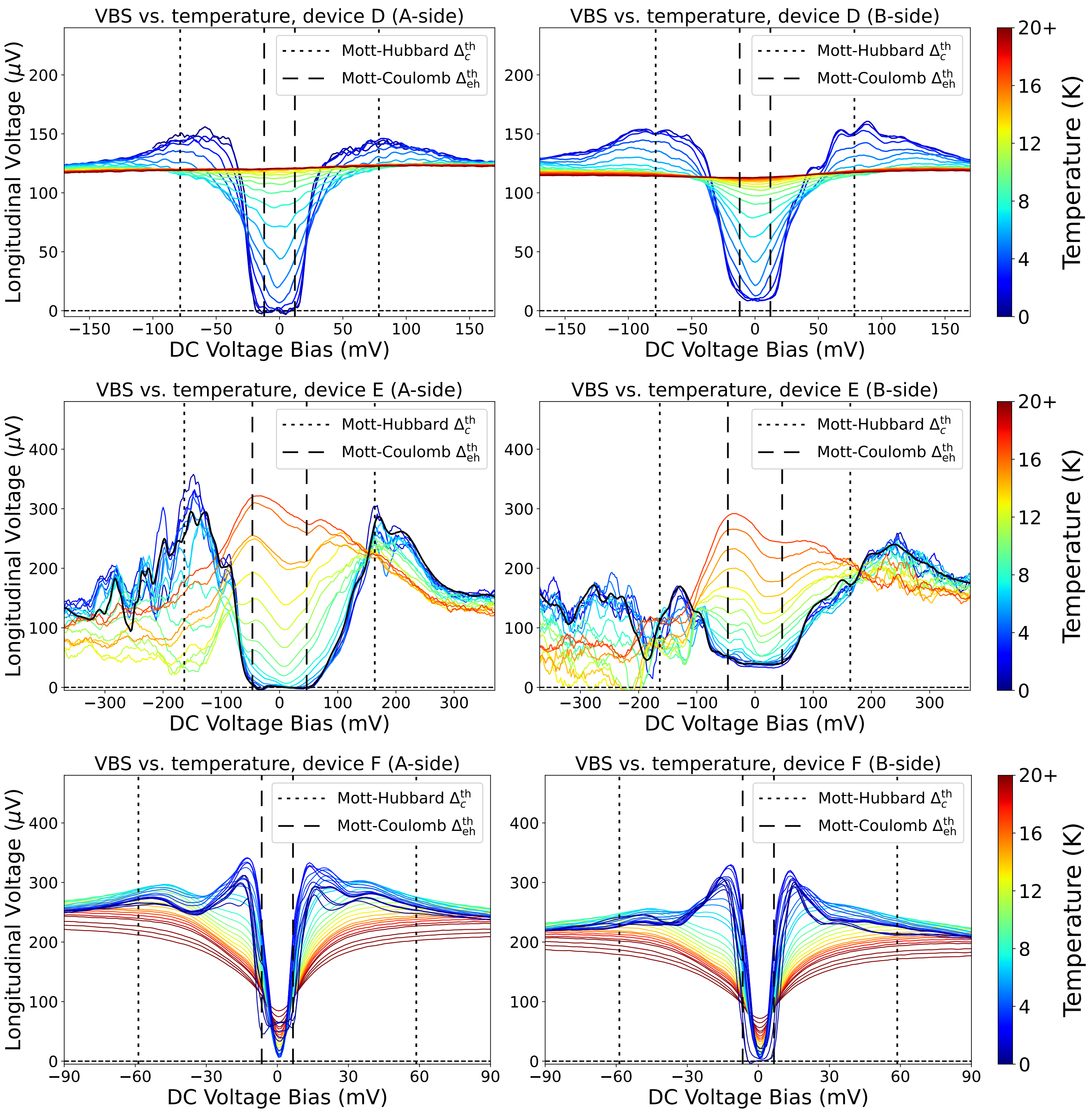

FIG. S16. **Thermal activation of voltage-bias spectroscopy for devices D, E, and F (top, centre, bottom row).** Successive voltage-bias spectroscopy (VBS) data are taken at increasing temperatures up to and exceeding $T = 20\,\text{K}$, with a shared color code for all plots. For each device, we show the longitudinal voltage measured along either side of the Hall bar (A/B-side, left and right plots); for devices D and F we find a good agreement between the measured VBS signatures on both sides, instilling confidence that observed signatures are due to bulk effects of the QD arrays rather than due to contacts or local disorder. VBS data for devices D and F were taken in a symmetrically-biased configuration, with source/drain voltages $V_{S/D} = \pm\frac{1}{2}V_{\text{DC}}$, while data for device E was taken with an asymmetric bias $V_S = V_{\text{DC}}$ and $V_D = 0$. Discrepancies in the VBS data for the A/B-side of the Hall bar in device E, and for positive/negative DC voltage bias, are discussed in the text. For each device the vertical dashed line shows the Mott-Coulomb particle-hole excitation gap $\Delta_{\text{eh}}^{\text{th}}$, and the vertical dotted line shows twice the Mott-Hubbard single-particle gap Δ_c^{th}, i.e., where the individual source/drain-to-device bias $V_{S/D}$ suffices to overcome the Mott-Hubbard gap Δ_c^{th}. For device E we also show the low-temperature VBS signal averaged across a magnetic field sweep (black line), see Sec. S4 D and Fig. S17. For further details and discussion, see Sec. S4 C.

individual quantum dots, and long-range interactions and screening in the arrays at scale, are important (cf. Sec. S3).

For device F, with the highest inter-dot conductance g_T among the insulating devices, in Fig. S16 (bottom row) we observe a strong coherence peak in the VBS signal (many available current-carrying states) just above the Coulomb gap scale $\Delta_{\mathrm{eh}}^{\mathrm{th}}$. As the temperature is raised the peak rapidly diminishes, and at all temperatures the VBS signal saturates once the applied DC voltage bias exceeds twice the predicted Mott-Hubbard gap, $|V_{\mathrm{dc}}| \gtrsim 2\Delta_{\mathrm{c}}^{\mathrm{th}}$. At this large bias, charge carriers in the source/drain (at voltage $V_{S/D} = \pm\frac{1}{2}V_{\mathrm{dc}}$) have sufficient energy to enter the dot array in spite of the single-particle charge gap $\Delta_{\mathrm{c}}^{\mathrm{th}}$. The suppression of charge transport (depletion of the VBS signal) in the region $|V_{\mathrm{dc}}| \lesssim 2\Delta_{\mathrm{c}}^{\mathrm{th}}$ appears to be more robust to increasing temperature than the hard Coulomb gap at $|V_{\mathrm{dc}}| \leq \Delta_{\mathrm{eh}}^{\mathrm{th}}$. This observation can be rationalized by the single- and two-particle nature of the underlying excitations, which may lead to a different thermal stability of the gap mechanisms and their signatures in charge transport. Importantly, all VBS signatures mentioned here are reproduced with close qualitative and near-quantitative agreement on both sides of the Hall bar (left/right plots in Fig. S16). This strongly supports our interpretation of these features as stemming from bulk physical effects in a roughly homogeneous (though locally disordered) quantum dot array, compared to a picture of large-scale disorder or contact effects in a macroscopically inhomogeneous system. Finally, for the magnetic-field tuned VBS in Fig. S17 (bottom) measured at base temperature and with lower AC probe bias, we resolve a weak but clearly visible secondary coherence peak at the Mott-Hubbard excitation energy scale $V_{S/D} = \pm\Delta_{c}^{\mathrm{th}}$.

For device D, with an intermediate inter-dot conductance g_T, in Fig. S16 (top row) we likewise observe a strongly suppressed VBS signal in the Coulomb gap $|V_{\mathrm{dc}}| \lesssim \Delta_{\mathrm{eh}}^{\mathrm{th}}$. From here the signal rises sharply, and at a source-drain bias $V_{\mathrm{dc}} \approx \pm 2\Delta_{c}^{\mathrm{th}}$ we observe a coherence peak in the low-temperature VBS that indicates many current-carrying states when the individual source/drain voltage $V_{S/D} = \pm\frac{1}{2}V_{\mathrm{dc}}$ suffices to inject charge carriers into the array from either contact. At elevated temperatures this coherence peak is rapidly suppressed, and the VBS signal is saturated for $|V_{\mathrm{dc}}| > 2\Delta_{c}^{\mathrm{th}}$; this is consistent with the source/drain bias overcoming the single-particle Mott-Hubbard gap, thus allowing unimpeded charge transport through the device. Note that the saturation of the thermal activation in VBS data for device D occurs more rapidly than in devices F and E, where already at $T \sim 15\,\mathrm{K}$ the signatures of a charge transport gap disappear. We surmise that this distinct thermal activation in voltage-bias spectroscopy may be related to the apparent lack of a coherent-electron regime in the low-bias thermal activation of conductance, cf. Supplementary Section S4 B and Fig. 4d of the main text. Also in device D all VBS signatures are reproduced with near-quantitative agreement for measurements taken on both sides of the Hall bar, indicating that they are due to bulk physics. The small offset of the VBS signal on the B-side (at low temperatures) can be traced back to coupling between wires that deliver the source-drain AC probe voltage and those attached to Hall bar contacts (or a similar effect elsewhere on the chip or circuit board); the offset interpolates to zero as the AC probe voltage is reduced, and merely represents a measurement artifact. The same effect occurs in device E, and less so in device F.

For device E, with the smallest inter-dot conductance g_T and strongest interactions, the VBS data in Fig. S16 (centre row) is more complicated to analyse. We have found that the drain contact of the Hall bar is not well connected, leading to significant changes in its behaviour as a function of temperature, especially considering the large source-drain biases $|V_{S/D}| \leq 0.5\,\mathrm{V}$ involved in the VBS experiment. This insight is based on the VBS data in Fig. S16 and further (a)symmetrically voltage- and current-biased experiments (not shown). We strongly suspect that in the VBS measurements of Fig. S16 a temperature-dependent fraction of the current entering from the source contact is drained either through the actual drain contact, or via different Hall bar side contacts. Since the Hall probes themselves have varying contact resistances, whenever a current is drawn through them the Hall probe voltage(s) at different contacts become temperature-dependent and asymmetric on A/B-sides of the Hall bar. This explains part of the mismatch between the A/B-side VBS in Fig. S16, and the asymmetry with respect to DC voltage bias. Further for the VBS measurements in this device, we have applied a positive top gate voltage $V_g \approx 0.7\,\mathrm{V}$ that allows us to make the quantum dot array a bit more conductive, i.e., to increase the inter-dot tunnelling t and conductance g_T, cf. Sec. S4 A-S4 B. For VBS at zero top-gate voltage $V_g = 0\,\mathrm{V}$ we have found the features discussed below are suppressed, likely due to the weak tunnelling and a partial, disorder-driven electron localization in the array. However working with a finite applied top-gate bias may lead to further shifts in the VBS features as the temperature is increased, since the effectiveness of the gate and the charge compressibility of the device are temperature-dependent.
For the discussion of device E we now focus on the VBS signal at the A-side of the Hall bar, since at low temperatures it is approximately symmetric about zero voltage bias, and at large positive bias $V_{\mathrm{dc}} \gtrsim 200\,\mathrm{mV}$ approaches a saturation value similar to what is observed in devices D and F. We find a strongly depleted charge transport regime at low bias within the Mott-Coulomb gap $|V_{\mathrm{dc}}| \lesssim \Delta_{\mathrm{eh}}^{\mathrm{th}}$, followed by a gradual raise to a pronounced coherence peak that matches the Mott-Hubbard excitation energy $V_{\mathrm{dc}} \approx \pm 2\Delta_{\mathrm{c}}^{\mathrm{th}}$. Finally, at large bias voltages $V_{\mathrm{dc}} > 2\Delta_{c}^{\mathrm{th}}$, the VBS signal saturates to an approximately temperature-independent value (also for the B-side VBS, at positive bias). The Mott-Coulomb and -Hubbard gap predicted from a pure electrostatic modelling of the device underestimate both of

these energy scales, cf. Table S1 and Sec. S3. The revised interaction and gap energy scales (used to plot $\Delta_c^{\rm th}$ and $\Delta_{\rm eh}^{\rm th}$ for Device E in Figure S16) based on the more accurate self-consistent field Hartree-Fock approach [21, 22] instead closely match the observed features in Fig. S16.

D. Exchange-interaction enhancement of the charge transport gap

A complementary tool to investigate correlated electron physics in our devices is the measurement of the charge transport gap Δ_c, from voltage-bias spectroscopy (VBS) of the longitudinal voltage as in Fig. 4b and S16, under an applied magnetic field B. The experiments and analysis presented here focus on the insulating devices D-F. The VBS data in Fig. S17 shows that the charge gap is enhanced in increasing magnetic field, following a roughly linear trend. The gap size change versus magnetic field extracted from the VBS data, see the right-hand plots in Fig. S17, is reproduced in Fig. 4c of the main text. The observed effect persists in parallel and perpendicular fields, pointing to the electron spin Zeeman effect as a likely origin, in contrast to magneto-orbital physics. We hence note

$$\Delta_c(B) \simeq \Delta_c^0 + \frac{1}{2} g_{\rm eff} \mu_B B \ , \quad g_{\rm eff} = g_0(1+\chi_s)$$

where the latter contribution to the gap size $\Delta_c(B)$ takes the form of a Zeeman energy, with the effective electron Lande g-factor $g_{\rm eff}$ extracted for each device. From the data in Fig. S17 we find $g_{\rm eff} \gg g_0 = 2$ that is larger in devices with smaller dot area A and stronger on-site interaction U. An enhanced g-factor in quantum dots is known to originate from the electron exchange interaction [35], which leads us to define the 'excess' spin susceptibility χ_s above [35, 36]. For the three insulating devices, we find

device	Δ_c^0	$g_{\rm eff}$	χ_s	$\chi_s/U\ \left[\frac{1}{\rm meV}\right]$	$\chi_s \cdot A\ \left[{\rm nm}^2\right]$
D	18.2	20.4	9.20	0.44	260
E	58.2	28.8	13.4	0.28	119
F	4.53	18.7	8.35	0.56	519

For a discussion of the zero-field VBS and charge transport gap Δ_c^0, see Sec. S4 C. For the magnetic-field dependence of the VBS, shown in Fig. S17 and summarized in Figs. 4c and 4e of the main text, the susceptibility ratio χ_s/U is very similar in the different devices D, E, and F. Note that the exact values $g_{\rm eff}$ and χ_s extracted from the VBS data depend on how the linear fit in Fig. S17 is performed, but in any case we obtain a large, interaction enhanced g-factor $g_{\rm eff} \gg g_0 = 2$ with $\chi_s \gg 1$. The scaling $\chi_s \sim U$ is expected for the exchange interaction in small quantum dots (or disordered metals) [35–38], while the precise form depends on the details of the material system. For example, the susceptibility may depend weakly on the inter-dot conductance and density of states [36] thus leading to relatively similar values χ_s/U for the different devices, or more strongly on the geometry of the quantum dot as $\chi_s \sim 1/A$ [37]. In either case the relatively large number of donors (active sites) within the dot (interacting region) allows the electrons to avoid part of the exchange energy cost. The fact that we observe this effect suggests that the electron wave-functions of typical orbital states in each quantum dot are spread across an extensive set of the dots' donor atoms. The latter property is highly desirable as it opens the possibility to design quantum dot and lattice geometries to promote specific electron orbital eigenstates and -energies, and inter-dot tunnelling amplitudes.

E. Low-temperature transport and Hall coefficient

Magneto-transport measurements of the longitudinal and transverse Hall conductances in the Hall bar setup of Fig. 1 (main text) allow us to further characterize the nature of electron conduction and the resulting low-temperature physics in our quantum dot arrays. We here focus primarily on the conductive samples A-C, since at present we were unable to obtain reliable low-temperature Hall coefficient data for the highly resistive devices D-F. The primary metric considered here is the temperature-dependent Hall coefficient $R_H(T)$, obtained as:

$$R_H = \left.\frac{d\rho_H}{dB}\right|_{B=0}$$

from the transverse Hall resistivity $\rho_H = \rho_{xy}$ (see Section S2 B for further discussion and details of the experiment). For weakly interacting electrons with a well-formed Fermi surface, $n_H = -1/eR_H$ measures the charge carrier density. As temperature is lowered, electrons gradually freeze out and localize due to disorder and interactions, leading to a

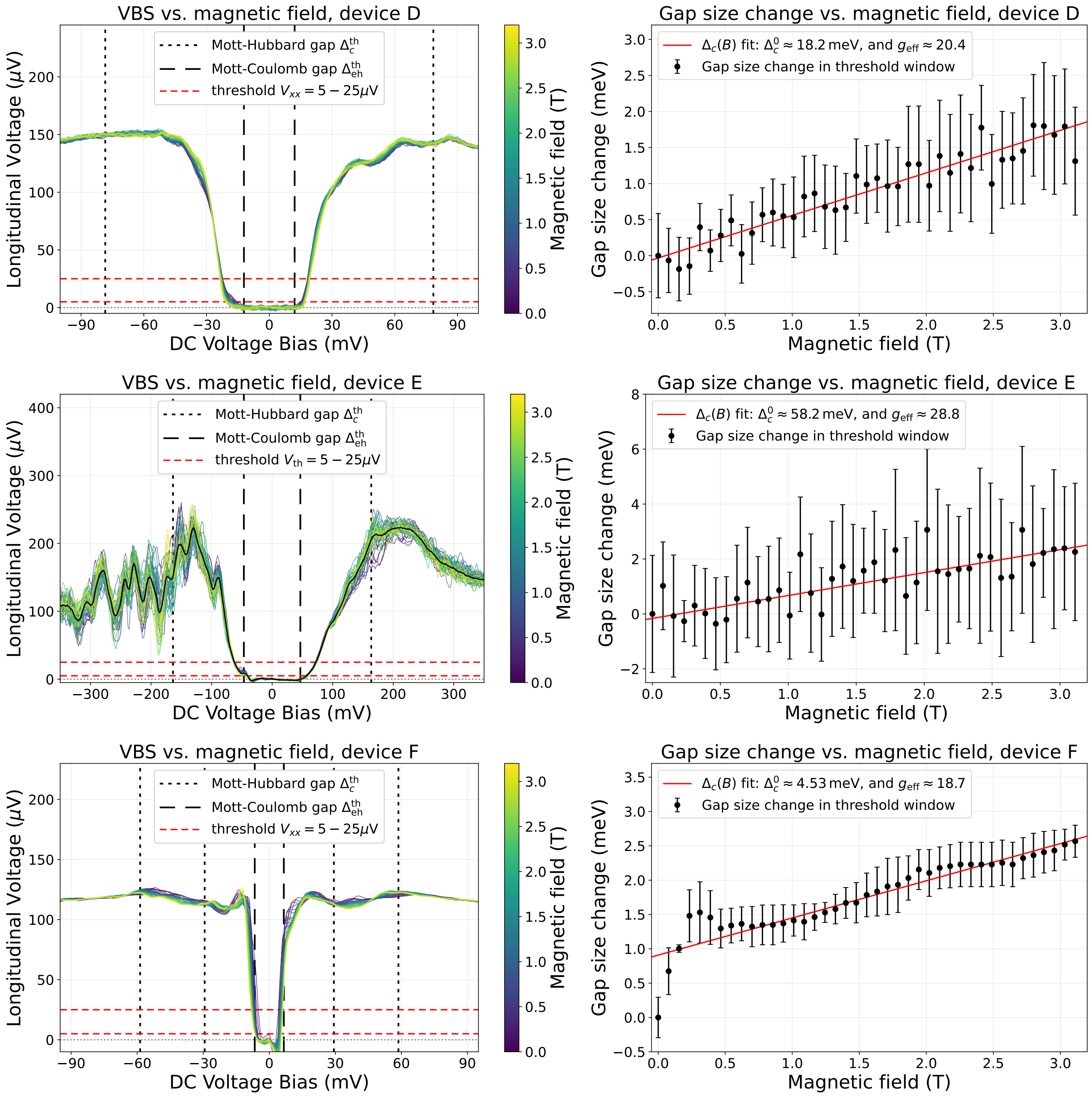

FIG. S17. **Magnetic-field dependence of voltage-bias spectroscopy (VBS) for Devices D, E, and F (top, centre, and bottom row).** Black dashed/dotted vertical lines are as for the thermal activation of VBS Fig. S16, and the VBS data is averaged for the A/B-sides of the Hall bar (zero-bias offset subtracted). For each device, from the VBS data (left) as function of magnetic field B (color-scale) we extract the charge gap $\Delta_c(B)$, sampled in the threshold window $V_{\rm th}$ (red dashed lines). A linear fit to the gap size change $\Delta_c(B) - \Delta_c^0 = \frac{1}{2} g_{\rm eff} \mu_B B$ (right) yields the zero-field gap Δ_c^0 and effective Lande g-factor $g_{\rm eff}$, quoted in the fit legend (red line). Note that while the exact results depend on the choice of threshold window, both the zero-field gap and effective g-factor are larger the more strongly interacting the device, and the latter is significantly larger than the bare electron g-factor, $g_{\rm eff} \gg g_0 = 2$. The VBS data are qualitatively reproduced in perpendicular and parallel magnetic fields, suggesting an electron spin Zeeman effect rather than magneto-orbital physics as origin of the field-driven enhancement of the charge gap. In these measurements device F was not perfectly thermally equilibrated at the start of the magnetic field sweep ($B \approx$ 0-0.4 T), hence the rapid initial change of the measured charge gap. For further discussion, see Sec. S4 D.

logarithmic decrease of $n_H(T)$ [8–11, 32]. This is observed in fully doped Si:P delta-layer samples [6, 29, 30], similar to our reference device (Supplementary S2 and Fig. S1). In artificial donor super-lattices, such as the devices in Fig. 1 and 2 of the main text, a reconfiguration of charge transport instead may occur due to a mini-band formation in the quantum dot array similar to what happens in moire materials [39–42]. Finally, in the Fermi-Hubbard model the Hall coefficient $R_H(T)$ is predicted to reveal a Fermi surface reconstruction as the hallmark of strongly-correlated physics [43–51]. Electrons form a coherent many-body state, and with lowering temperature the nature of charge transport and hence $R_H(T)$ is drastically altered, even though the number of dopants in the device remains unchanged and only a single band is considered. In our devices we expect a combination of electron confinement in the donor quantum dot lattice (mini-band formation), disorder (electron localization), and strong interactions (Fermi-Hubbard physics) to determine the charge transport and Hall coefficient. Which of these is the dominant mechanism at what temperatures is near impossible to disentangle, at present, without a full microscopic model of the devices.
The parameters that control the Hall coefficient are the interaction strengths U, V and tunnelling t, and the cross-over temperature T_c. To observe effects driven by coherent tunnelling requires $T < T_c$. In strongly-interacting quantum dot arrays, the mini-band structure and many-body Fermi-Hubbard phenomenology then develops at and below the scale $k_B T_t = t$ [39–42, 47–50], with another pronounced change once temperature approaches the exchange energy $k_B T_J = J_{\text{exc}} \simeq 4t^2/(U - V)$ [47, 51, 52]. For the present devices, we estimate these values as

device	A	B	C	D	E	F
T_c[K]	22.6	18.1	17.1	8.98	29.0	5.47
T_t[K]	17.9	9.60	5.66	1.19	1.38	0.72
T_J[K]	6.87	2.02	0.66	0.03	0.02	0.01

Measurements of the Hall coefficient for samples A-C are shown in Fig. 5 of the main text, together with a discussion in the framework of the above physical effects and temperature scales. An extensive discussion of the magneto-transport measurements and the full source data and analysis is provided in Sec. S2 B and Figs. S2-S14.

[1] T.-C. Shen, C. Wang, G. Abeln, J. Tucker, J. Lyding, P. Avouris, and R. Walkup, Atomic-scale desorption through electronic and vibrational excitation mechanisms, Science **268**, 1590 (1995).

[2] J. Lyding, T.-C. Shen, J. Hubacek, J. Tucker, and G. Abeln, Nanoscale patterning and oxidation of H-passivated Si (100)-2× 1 surfaces with an ultrahigh vacuum scanning tunneling microscope, Applied Physics Letters **64**, 2010 (1994).

[3] F. J. Rueß, W. Pok, T. C. Reusch, M. J. Butcher, K. E. J. Goh, L. Oberbeck, G. Scappucci, A. R. Hamilton, and M. Y. Simmons, Realization of atomically controlled dopant devices in silicon, Small **3**, 563 (2007).

[4] M. Simmons, F. Ruess, K. Goh, W. Pok, T. Hallam, M. Butcher, T. Reusch, G. Scappucci, A. Hamilton, and L. Oberbeck, Atomic-scale silicon device fabrication, International Journal of Nanotechnology **5**, 352 (2008).

[5] S. W. Schmucker, P. N. Namboodiri, R. Kashid, X. Wang, B. Hu, J. E. Wyrick, A. F. Myers, J. D. Schumacher, R. M. Silver, and M. D. Stewart, Low-resistance, high-yield electrical contacts to atom scale si:p devices using palladium silicide, Phys. Rev. Appl. **11**, 034071 (2019).

[6] S. R. McKibbin, W. R. Clarke, and M. Y. Simmons, Investigating the surface quality and confinement of Si:P δ -layers at different growth temperatures, Physica E: Low-Dimensional Systems and Nanostructures **42**, 1180 (2010).

[7] S. Hikami, A. I. Larkin, and Y. Nagaoka, Spin-orbit interaction and magnetoresistance in the two dimensional random system, Progress of Theoretical Physics **63**, 707 (1980).

[8] D. Belitz and T. R. Kirkpatrick, The Anderson-Mott transition, Rev. Mod. Phys. **66**, 261 (1994).

[9] P. A. Lee and T. V. Ramakrishnan, Disordered electronic systems, Rev. Mod. Phys. **57**, 287 (1985).

[10] M. Imada, A. Fujimori, and Y. Tokura, Metal-insulator transitions, Rev. Mod. Phys. **70**, 1039 (1998).

[11] I. S. Beloborodov, A. V. Lopatin, V. M. Vinokur, and K. B. Efetov, Granular electronic systems, Rev. Mod. Phys. **79**, 469 (2007).

[12] I. S. Beloborodov, A. V. Lopatin, and V. M. Vinokur, Coulomb effects and hopping transport in granular metals, Phys. Rev. B **72**, 125121 (2005).

[13] M. Y. Kharitonov and K. B. Efetov, Hall resistivity of granular metals, Phys. Rev. Lett. **99**, 056803 (2007).

[14] C. Multiphysics, Introduction to comsol multiphysics®, COMSOL Multiphysics, Burlington, MA, accessed Feb **9**, 2018 (1998).

[15] X. Wang, E. Khatami, F. Fei, J. Wyrick, P. Namboodiri, R. Kashid, A. F. Rigosi, G. Bryant, and R. Silver, Experimental realization of an extended Fermi-Hubbard model using a 2D lattice of dopant-based quantum dots, Nature Communications **13**, 6824 (2022).

[16] J. Maxwell, *A Treatise on Electricity and Magnetism*, A Treatise on Electricity and Magnetism No. v. 1 (Clarendon Press, 1873).

[17] W. G. Van der Wiel, S. De Franceschi, J. M. Elzerman, T. Fujisawa, S. Tarucha, and L. P. Kouwenhoven, Electron transport through double quantum dots, Reviews of modern physics **75**, 1 (2002).

[18] L. P. Kouwenhoven, D. Austing, and S. Tarucha, Few-electron quantum dots, Reports on progress in physics **64**, 701 (2001).
[19] O. Ciftja, Coulomb self-energy and electrostatic potential of a uniformly charged square in two dimensions, Physics Letters A **374**, 981 (2010).
[20] O. Ciftja, Hartree–fock energy of a finite two-dimensional electron gas system in a jellium background, Physica B: Condensed Matter **458**, 92 (2015).
[21] A. M. S.-E. Huq, *Designing High-performance Qubits using Dopant based Quantum Dots*, Ph.D. thesis, UNSW Sydney (2023).
[22] S. Ahmed, N. Kharche, R. Rahman, M. Usman, S. Lee, H. Ryu, H. Bae, S. Clark, B. Haley, and M. Naumov, Multimillion atom simulations with NEMO 3-D, arXiv preprint arXiv:0901.1890 (2009).
[23] N. H. Le, A. J. Fisher, and E. Ginossar, Extended Hubbard model for mesoscopic transport in donor arrays in silicon, Phys. Rev. B **96**, 245406 (2017).
[24] A. Dusko, A. Delgado, A. Saraiva, and B. Koiller, Adequacy of Si:P chains as Fermi-Hubbard simulators, npj Quantum Information **4**, 1 (2018).
[25] H. Ryu, S. Lee, B. Weber, S. Mahapatra, L. C. Hollenberg, M. Y. Simmons, and G. Klimeck, Atomistic modeling of metallic nanowires in silicon, Nanoscale **5**, 8666 (2013).
[26] M. B. Donnelly, M. M. Munia, J. G. Keizer, Y. Chung, A. S.-E. Huq, E. N. Osika, Y.-L. Hsueh, R. Rahman, and M. Y. Simmons, Multi-scale modeling of tunneling in nanoscale atomically precise Si:P tunnel junctions, Advanced Functional Materials **33**, 2214011 (2023).
[27] P. Barthelemy and L. M. K. Vandersypen, Quantum dot systems: a versatile platform for quantum simulations, Annalen der Physik **525**, 808 (2013).
[28] E. Barnes, J. P. Kestner, N. T. T. Nguyen, and S. Das Sarma, Screening of charged impurities with multielectron singlet-triplet spin qubits in quantum dots, Phys. Rev. B **84**, 235309 (2011).
[29] K. E. J. Goh, L. Oberbeck, M. Y. Simmons, A. R. Hamilton, and M. J. Butcher, Influence of doping density on electronic transport in degenerate si:p δ-doped layers, Phys. Rev. B **73**, 035401 (2006).
[30] K. E. J. Goh, M. Y. Simmons, and A. R. Hamilton, Electron-electron interactions in highly disordered two-dimensional systems, Phys. Rev. B **77**, 235410 (2008).
[31] B. I. Shklovskii and A. L. Efros, *Electronic properties of doped semiconductors*, Vol. 45 (Springer Science & Business Media, 2013).
[32] I. S. Beloborodov, K. B. Efetov, A. V. Lopatin, and V. M. Vinokur, Transport properties of granular metals at low temperatures, Phys. Rev. Lett. **91**, 246801 (2003).
[33] M. V. Feigel'man and A. S. Ioselevich, Variable-range cotunneling and conductivity of a granular metal, JETP Letters **81**, 277 (2005).
[34] A. L. Efros and B. I. Shklovskii, Coulomb gap and low temperature conductivity of disordered systems, Journal of Physics C: Solid State Physics **8**, L49 (1975).
[35] Y. Alhassid, The statistical theory of quantum dots, Rev. Mod. Phys. **72**, 895 (2000).
[36] A. V. Andreev and A. Kamenev, Itinerant ferromagnetism in disordered metals: A mean-field theory, Phys. Rev. Lett. **81**, 3199 (1998).
[37] P. W. Brouwer, Y. Oreg, and B. I. Halperin, Mesoscopic fluctuations of the ground-state spin of a small metal particle, Phys. Rev. B **60**, R13977 (1999).
[38] Y. M. Blanter, A. D. Mirlin, and B. A. Muzykantskii, Fluctuations of conductance peak spacings in the coulomb blockade regime: Role of electron-electron interaction, Phys. Rev. Lett. **78**, 2449 (1997).
[39] D. M. Kennes, M. Claassen, L. Xian, A. Georges, A. J. Millis, J. Hone, C. R. Dean, D. Basov, A. N. Pasupathy, and A. Rubio, Moiré heterostructures as a condensed-matter quantum simulator, Nature Physics **17**, 155 (2021).
[40] Y. Cao, V. Fatemi, S. Fang, K. Watanabe, T. Taniguchi, E. Kaxiras, and P. Jarillo-Herrero, Unconventional superconductivity in magic-angle graphene superlattices, Nature **556**, 43 (2018).
[41] Y. Cao, V. Fatemi, A. Demir, S. Fang, S. L. Tomarken, J. Y. Luo, J. D. Sanchez-Yamagishi, K. Watanabe, T. Taniguchi, E. Kaxiras, R. C. Ashoori, and P. Jarillo-Herrero, Correlated insulator behaviour at half-filling in magic-angle graphene superlattices, Nature **556**, 80 (2018).
[42] Y. Tang, L. Li, T. Li, Y. Xu, S. Liu, K. Barmak, K. Watanabe, T. Taniguchi, A. H. MacDonald, J. Shan, *et al.*, Simulation of Hubbard model physics in WSe_2/WS_2 Moiré superlattices, Nature **579**, 353 (2020).
[43] M. Qin, T. Schäfer, S. Andergassen, P. Corboz, and E. Gull, The Hubbard model: a computational perspective, Annual Review of Condensed Matter Physics **13**, 275 (2022).
[44] D. P. Arovas, E. Berg, S. A. Kivelson, and S. Raghu, The Hubbard model, Annual Review of Condensed Matter Physics **13**, 239 (2022).
[45] S. Nair, S. Wirth, S. Friedemann, F. Steglich, Q. Si, and A. J. Schofield, Hall effect in heavy Fermion metals, Advances in Physics **61**, 583 (2012).
[46] C. Proust and L. Taillefer, The remarkable underlying ground states of cuprate superconductors, Annual Review of Condensed Matter Physics **10**, 409 (2019).
[47] W. O. Wang, J. K. Ding, B. Moritz, E. W. Huang, and T. P. Devereaux, DC Hall coefficient of the strongly correlated hubbard model, npj Quantum Materials **5**, 51 (2020).
[48] W. O. Wang, J. K. Ding, B. Moritz, Y. Schattner, E. W. Huang, and T. P. Devereaux, Numerical approaches for calculating the low-field DC Hall coefficient of the doped Hubbard model, Phys. Rev. Res. **3**, 033033 (2021).
[49] I. Khait, S. Bhattacharyya, A. Samanta, and A. Auerbach, Hall anomalies of the doped mott insulator, npj Quantum

Materials **8**, 75 (2023).
[50] F. F. Assaad and M. Imada, Hall coefficient for the two-dimensional Hubbard model, Phys. Rev. Lett. **74**, 3868 (1995).
[51] B. S. Shastry, B. I. Shraiman, and R. R. P. Singh, Faraday rotation and the hall constant in strongly correlated fermi systems, Phys. Rev. Lett. **70**, 2004 (1993).
[52] P. G. J. van Dongen, Extended hubbard model at strong coupling, Phys. Rev. B **49**, 7904 (1994).
